# Comparison of Methods for Estimating Dietary Food and Nutrient Intakes and Intake Densities from Household Consumption and Expenditure Data in Mongolia

**DOI:** 10.3390/nu10060703

**Published:** 2018-05-31

**Authors:** Sabri Bromage, Bernard Rosner, Janet W. Rich-Edwards, Davaasambuu Ganmaa, Soninkhishig Tsolmon, Zuunnast Tserendejid, Tseye-Oidov Odbayar, Margaret Traeger, Wafaie W. Fawzi

**Affiliations:** 1 Department of Nutrition, Harvard T.H. Chan School of Public Health, Harvard University, Boston, MA 02115, USA; 2 Department of Biostatistics, Harvard T.H. Chan School of Public Health, Harvard University, Boston, MA 02115, USA; stbar@channing.harvard.edu; 3 Connors Center for Women’s Health and Gender Biology, Brigham and Women’s Hospital, Harvard Medical School, Boston, MA 02115, USA; jwrichedwards@bics.bwh.harvard.edu; 4 Channing Division of Network Medicine, Department of Medicine, Brigham and Women’s Hospital and Harvard Medical School, Boston, MA 02115, USA; gdavaasa@hsph.harvard.edu; 5 Department of Nutrition, Mongolian University of Science and Technology, Ulaanbaatar 14191, Mongolia; soninkhishig@must.edu.mn (S.T.); znast_03@yahoo.com (Z.T.); 6 Department of Food Engineering, Mongolian University of Science and Technology, Ulaanbaatar 14191, Mongolia; odbayar_ts@must.edu.mn; 7 Department of Sociology, Yale University, New Haven, CT 06511, USA; margaret.traeger@yale.edu; 8 Department of Global Health and Populations, Harvard University T.H. Chan School of Public Health, Boston, MA 02115, USA; mina@hsph.harvard.edu

**Keywords:** household consumption and expenditure, adult male equivalent, intra-household distribution, nutrient density, dietary survey, nutrition surveillance, Mongolia

## Abstract

Household consumption and expenditure surveys are frequently conducted around the world and they usually include data on household food consumption, but their applicability to nutrition research is limited by their collection at the household level. Using data from Mongolia, this study evaluated four approaches for estimating diet from household surveys: direct inference from per-capita household consumption; disaggregation of household consumption using a statistical method and the “adult male equivalent” method, and direct prediction of dietary intake. Per-capita household consumption overestimated dietary energy in single- and multi-person households by factors of 2.63 and 1.89, respectively. Performance of disaggregation methods was variable across two household surveys analyzed, while the statistical method exhibited less bias in estimating intake densities (per 100 kcal) of most dietary components in both of the surveys. Increasingly complex prediction models explained 54% to 72% of in-sample variation in dietary energy, with consistent benefits incurred by inclusion of basic dietary measurements. In conclusion, in Mongolia and elsewhere, differences in how household and dietary measurements are recorded make their comparison challenging. Validity of disaggregation methods depends on household survey characteristics and the dietary components that are considered. Relatively precise prediction models of dietary intake can be achieved by integrating basic dietary assessment into household surveys.

## 1. Introduction

Low coverage, frequency, and quality of dietary data from industrializing populations are significant obstacles in understanding diet-disease relationships and designing effective nutrition policies and programs around the world [1]. While developed countries often benefit from large, periodic surveys that collect 24-h recall, diet record, or food-frequency data [2], dietary surveys in developing countries typically employ less rigorous methodologies, owing to the resources required for hiring dieticians or training community health workers, collecting dietary measurements from hundreds or thousands of people, and compiling local food composition data of adequate expansiveness [3,4]. To save money, nutrition surveys in developing countries sometimes collect data at the household—rather than the individual—(dietary-) level, which usually involves asking questions about household food security or dietary diversity [5,6]. Such data are suited to address key nutritional questions, but they do not allow for complete enumeration of household food or nutrient consumption, and the range of interventions that these data can inform is limited accordingly.

In contrast with household food security assessments, a more detailed class of household survey—the household consumption and expenditure (HCE) survey, frequently administered by national statistical offices—had been collected at least 700 times from 116 countries as of 2012 [7]. Along with many useful covariates, HCE data often contain information on recent household food consumption (collected using a household diary or recall instrument), and are relatively inexpensive to collect periodically [8]. While HCE data are more applicable to designing and evaluating nutrition interventions than food balance sheets [9], their limitations have been characterized extensively, as have the validity of household food consumption estimates [10]. A fundamental limitation of HCE data for nutrition research is that, with the exception of single-person households, they do not allow for the direct estimation of dietary intake by individuals, but of per-capita household consumption among different household strata (e.g., defined by region, season, or socio-economic status), which is useful for informing certain interventions, but not others [11]. Disaggregation of household food consumption to estimate individuals’ dietary intake (i.e., “individualization”) requires often tenuous assumptions, and it is frequently limited to screening for dietary intake deficiencies or estimating dietary intake of specific fortification vehicles rather than broader nutritional surveillance or epidemiology [12,13]. The former are important objectives in and of themselves, however.

One approach to disaggregating household consumption data, called the adult male equivalent (AME) or adult equivalent unit (AEU) method, is commonly used for estimating individuals’ dietary intake in the absence of dietary measurements [14,15]. The AME method divides consumption of household foods or nutrients (collected as part of an HCE survey) in a manner proportional to the predicted nutrient requirements of household members (usually energy requirements). For the AME method to be accurate, a primary assumption that must be met is that household distribution of consumed foods and nutrients is equitable with respect to household members’ caloric requirements. There is evidence that this assumption holds for certain demographics within certain national populations, but not others [16]. An alternative disaggregation method using regression was proposed by Chesher in 1997, which was based on work by Engle and colleagues [17,18], and has arisen in the literature sporadically since its conception. This statistical method involves an indirect inference of individuals’ dietary intake through the prediction of total household food consumption. Attempts have not been made to validate this method against dietary measurements or to compare its performance with the AME method.

In this study, we analyzed a combination of household food consumption and individual dietary measurements from Mongolia to assess the comparative usefulness of four approaches for applying household survey data to estimate dietary food and nutrient intake and intake density (i.e., intake per 100 kcal) in that country. Our four aims were the following (these four aims are referred to by number throughout this paper):

1. to compare (a) per-capita estimates of household food and nutrient consumption obtained from household-level measurements with (b) per-capita dietary measurements that were obtained from individuals in the same households;
2. to compare (a) estimates of individuals’ food and nutrient intake obtained by applying the AME disaggregation method to household consumption measurements with (b) direct measurements of dietary intake obtained from the same individuals;
3. to compare (a) estimates of individuals’ food and nutrient intake obtained by applying the statistical disaggregation method to household consumption measurements with (b) direct measurements of dietary intake obtained from the same individuals; and,
4. To evaluate the ability of household survey data to predict individuals’ dietary nutrient intake given the availability of (i) direct dietary measurements and (ii) a broad set of household- and individual level characteristics that were obtained from the same individuals.

Individuals’ dietary measurements analyzed in Aims 1 to 4 were collected using a multiple pass 24-h recall developed for Mongolian adults.

## 2. Materials and Methods

### 2.1. Sources of Household Food Consumption Data

Data from two nationally-representative household surveys in Mongolia were analyzed in this study: the 2013 Food Consumption Survey (FCS-HH) [19], which conducted by the Mongolian University of Science and Technology (*n* = 1017 households comprising 4087 individuals), and pooled 2012 and 2014 independently-sampled survey waves of the Household Socio-Economic Survey (HSES-HH), which was conducted by the National Statistics Office (Ulaanbaatar, Mongolia) (*n* = 28,985 households comprising 106,760 individuals) [20]. The FCS-HH and HSES-HH collected data on household consumption of 116 and 118 different foods, respectively, from three sources (foods produced, purchased, and received as gifts, only the sum of which was analyzed in the current study) over a recent reference period, using a recall instrument in the case of the FCS-HH and a daily diary in the HSES-HH. The reference period was defined as the past week or month (whichever was more convenient for the household’s enumerator to recall for each food) in the case of the FCS-HH, the past week in the case of rural households in the HSES-HH, and the past 10, 10 to 20, and 20 to 30 days in the case of urban households in the HSES-HH. (Throughout this paper, “urban” is used to refer to the capital municipality of Ulaanbaatar and province (aimag) and county (soum) centers, while “rural” refers to more remote settlements and the countryside). Although pooling data from all three HSES-HH reference periods would allow for more precise long-term estimates of household consumption, later periods (the last 20 days of record collection) have been observed to be more prone to underreporting in prior survey waves [21] (as we also found in exploratory analyses of the 2012–2014 waves), in part due to fatigue incurred by maintaining a diary of household consumption for an entire month. Considering this, this study analyzed HSES-HH household food consumption data from the earlier first 10 days only. HSES-HH waves were conducted year-round, while the FCS-HH was conducted from May to August; for comparability with the FCS-HH, analysis of HSES-HH data were thus restricted to 9849 households comprising 35,920 individuals from which data were collected between those months.

### 2.2. Sources of Dietary Intake Data

In addition to the aforementioned sources of household data, the current study also analyzed 24-h dietary recall (24HR) data collected from a subset of 1369 randomly-sampled individuals that were aged 15 years or older participating in the FCS (this nested dataset is referred to as the FCS-24). Dietary data were not collected from participants in the HSES. The 24HR consisted of a multiple pass method that was developed for Mongolian adults by dieticians at the University of Science and Technology (the method and its development are described in Appendix A). The recall data describe individuals’ dietary intakes of 160 distinct food items composed of 136 distinct ingredients. Nutritional analysis of the FCS-HH, nested FCS-24, and HSES-HH also drew upon ancillary data on food composition, cooking yields, and components of variance in dietary nutrient intake collected and analyzed from 2012 to 2016 as part of a separate nationwide dietary assessment, in which paired summer and winter three-day weighed diet records were collected 320 healthy Mongolian men and women aged 22–55 living in urban and rural areas of seven national provinces and Ulaanbaatar [22].

Participants of the FCS-HH and nested FCS-24, HSES-HH, and nationwide diet records surveys provided written informed consent prior to enrollment. Collection of these surveys’ data and their analysis in the current study was permitted by the ethics boards of the National Statistical Office of Mongolia, Mongolian University of Science and Technology, and Harvard University T.H. Chan School of Public Health, respectively.

### 2.3. Preparation of Data for Analysis

Foods across the FCS-HH, nested FCS-24, and HSES-HH were condensed into 11 food groups, ages were condensed into 10 age groups, and variables were created to describe individuals’ total daily predicted caloric requirement, each household’s fractions of total caloric requirements contributed by permanent and impermanent members, and household family composition category (Appendix B; Table S1). Survey weights were derived for the FCS-HH, nested FCS-24, and HSES-HH using the national census (Appendix C). Household food consumption in the FCS-HH and HSES-HH was converted to g/day and was adjusted for refuse, spoilage, waste, and eating out (Appendix D). Individuals’ daily dietary nutrient intake in the FCS-24, and households’ total daily nutrient consumption in the FCS-HH and HSES-HH were calculated using a purpose built food composition table incorporating locally-analyzed food samples and locally-collected recipes and yield factors; dietary intake and household consumption of food groups and nutrients were also expressed in “energy-adjusted” terms (per 100 kcal of intake or consumption, respectively) to produce “intake densities” and “consumption densities”, respectively (Appendix E). Individuals’ dietary nutrient intakes in the FCS-24 were adjusted for within-person variance using variance components that were estimated for the national population (Appendix F; Table S2).

### 2.4. Exclusion Criteria and Descriptive Statistics

Four hundred and thirteen households in the HSES-HH were excluded from further analyses for containing no permanent household members. Five and 16 households in the FCS-HH and HSES-HH, respectively, for which the ratio of calories in total household food consumption to the total predicted energy expenditure of household members lay three standard deviations beyond the median were further excluded, following a comparable approach for individuals in the literature [23]. One individual with no observed dietary intake was excluded from analysis of the FCS-24HR, and four were further excluded for having extreme ratios of daily total energy intake (TEI) to total energy expenditure (TEE) (> or <3SD) after adjustment for within-person variance. While such extreme values are not necessarily implausible for individuals given only one day of observed intake, their plausibility was considered less likely after adjustment for within-person variance. Such extreme values were generally considered as less plausible for households in the FCS-HH and HSES-HH given the length of the reference periods considered in the household surveys.

**Figure 1:**
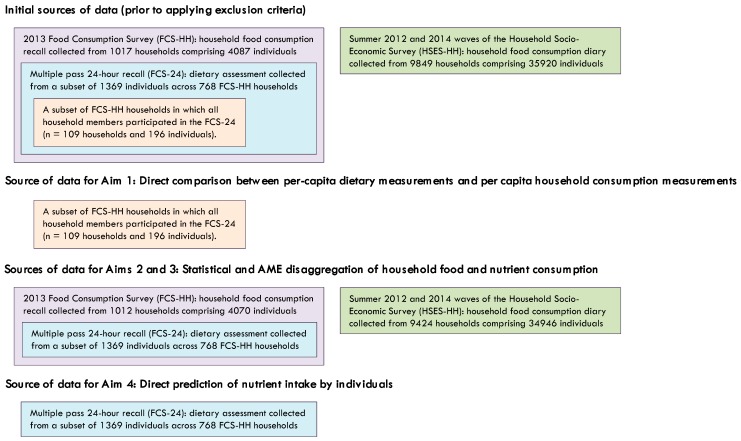
Sources of data. See Methods for description of exclusion criteria and information regarding ancillary diet records and national census data used in preparing and analyzing the 2013 Food Consumption Survey (FCS-HH), nested 2013 Food Consumption Survey-24-h (FCS-24), and Household Socio-Economic Survey (HSES-HH).

After applying exclusion criteria, 109 FCS-HH households were available, in which 24HR data had been collected from all of the household members, allowing for direct comparison between per-capita household consumption and per-capita dietary measurements from the same households (Aim 1); 9424 and 1012 households were available in the HSES-HH and FCS-HH, respectively, for the disaggregation of household food group and nutrient consumption to estimate dietary intakes of individuals (Aims 2 and 3); and, FCS-24 dietary data were available from 1356 individuals for comparison with disaggregated household consumption estimates (Aims 2 and 3) and prediction of individuals’ dietary nutrient intakes (Aim 4). A summary of the sources of data analyzed in each of the four Aims is provided in Figure 1. Demographic and socioeconomic characteristics of households and constituent household members in the FCS-HH and HSES-HH were tabulated after applying exclusion criteria (Table 1), as were the proportions of individuals in the FCS24 and households in the FCS-HH and HSES-HH observed to consume any of each food group or nutrient during each survey’s reference period and the correlations between the food groups and selected nutrients within both the FCS-HH and FCS-24 datasets.

### 2.5. Aim 1: Direct Comparison between Per-Capita Household Consumption and Per-Capita Dietary Measurements from the Same Households

An initial assessment of the comparability of individual dietary intake and household consumption measurements was made by considering the subset of109 FCS-HH households whose members were fully-enumerated by the nested FCS-24 (i.e., FCS-HH households for which 24HR measurements were collected for all individual household members, in addition to total household food consumption, as measured using the household recall instrument). For each of these 109 FCS-HH households, estimates of daily per-capita food group and nutrient consumption and consumption densities (consumption per 100 kcal) were derived from household food consumption measurements, as were per-capita dietary food group and nutrient intakes and intake densities that were derived from the sum of 24HR measurements collected from all household members. Among both single-person and multi-person households, mean per-capita household consumption and consumption density of each food group and nutrient was calculated from per-capita household consumption estimates, mean per-capita dietary intake, and intake density was calculated from per-capita dietary intake estimates, and mean difference, mean ratio, Pearson correlation coefficient, and Spearman rank correlation coefficient were calculated between paired per-capita household-derived and dietary-derived measurements from the same households. Calculation of mean ratios excluded a single two-person household with implausibly low per-capita household energy consumption (3 kcal/day).

### 2.6. Statistical Disaggregation of Household Food and Nutrient Consumption

Two disaggregation methods were applied to the FCS-HH and HSES-HH in an attempt to estimate dietary food group and nutrient intake by household members. First, a “statistical” disaggregation method was applied using generalized linear models (R v3.4 ‘glm’ package, IBM, Chicago, Illinois, USA) with a Tweedie response distribution and identity link function [24]. This allowed for a set of models that could flexibly accommodate both zero-inflated and right-skewed response data (issues in analysis of food groups, and both food groups and nutrients, respectively) and provided easily interpretable parameter estimates on the response scale. Total daily household consumption of each food group and nutrient was regressed in a survey-weighted model on 25 household variables, including a set of 20 integer variables collectively describing the number of household members of each age-sex group in each household, the fraction of household daily energy expenditure contributed by the person-time of impermanent members, family composition category, locality (urban, peri-urban/suburban, and rural), the fraction of household food spending on food eaten outside home, and the maximum number of years of education that was obtained by any household member (given Mongolia’s sizeable population of nomadic pastoralists, education is considered a more useful measure of socio-economic status than income [25]; adjustment for family composition and consumption by impermanent members generally follows methods described by Chesher [18]).

Models were fit for each of 21 possible values of the Tweedie index parameter *p* (ranging from 1 to 3 in increments of 0.1) to select the parameter value that produced the smallest ratio of residual to null deviance. Models were weighted using the survey weights previously described. The parameter estimates associated with each of the 10 age groups within each sex were extracted along with their respective 95% confidence limits to derive sex-specific age-intake relationships for each food group and nutrient, each of which was smoothed across the age groups using regression splines and a subjective smoothing parameter selected based on visual inspection (“gam” package) [26]. After smoothing, negative parameter estimates and confidence limits were adjusted to 0 for interpretability. Goodness of fit for each model was recorded in terms of the proportion of deviance explained (1-residual deviance/null deviance), associated Chi-square test *p*-value, and mean absolute error. For each age- and sex-group, disaggregated household consumption density estimates (estimated consumption per 100 kcal) were obtained by dividing the group’s disaggregated estimate of food group or nutrient consumption by its disaggregated estimate of energy consumption and then multiplying by 100. A similar approach to obtaining nutrient ratios by “manipulating estimates of underlying nutrient intakes” from a pair of statistical disaggregation models follows that first used by Chesher [18,27].

### 2.7. AME Disaggregation of Household Food and Nutrient Consumption

To obtain results that are more comparable with the statistical method’s, application of the adult male equivalent (AME) method was preceded by adjustment of daily household food group and nutrient consumption in the FCS-HH and HSES-HH for the variables family composition, household locality, fraction of outside food spending, maximum number of years of education attained, and fraction of household energy consumption attributed to the person time of impermanent members (variables also adjusted for in analysis using the statistical method). Adjustment proceeded using the residual method [28], in which household food group or nutrient consumption was regressed in a linear model upon the four predictors, and the residuals were extracted and scaled by adding to them the mean of household consumption across all households, producing residual-adjusted measures of daily household consumption (negative values of household consumption resulting from this adjustment were set to 0 for interpretability). The AME method was then applied to each household survey by multiplying each household’s residual-adjusted food group and nutrient consumption by the household’s total caloric requirement. Disaggregated estimates of food and nutrient consumption density were derived by dividing each household member’s AME-disaggregated food or nutrient consumption estimates by their AME-disaggregated energy consumption estimate and then multiplying by 100. A survey-weighted mean of consumption and consumption density of each food group and nutrient was then computed within each age group and sex (in the case of nutrient densities, a trimmed mean was taken to provide results robust to extreme ratios). Disaggregated household consumption estimates were smoothed across the 10 age groups within each sex using the same approach as in the statistical method.

### 2.8. Comparison between Disaggregated Household Consumption Estimates and Individual Dietary Intake Measurements (Aims 2 and 3)

The validity of each disaggregation method was evaluated based on its ability to estimate dietary food group and nutrient intake and intake densities of individuals by comparing disaggregated household consumption estimates from the HSES-HH and FCS-HH with within-person variance-adjusted individual dietary measurements from the nested FCS-24. Three validation metrics (bias, coverage probability, and ability to rank consumption) were derived for each disaggregation method (statistical and AME), food group and nutrient, class of estimate (consumption and consumption density), and household survey (FCS-HH and HSES-HH). While statistical- and AME-disaggregated household consumption estimates are computed using survey weights, the FCS-24 measurements are not weighted, therefore each of the three validation metrics implicitly account for survey weights and are nationally-representative statistics.

(1) Bias (observed—predicted value) was calculated for each of the 1356 individuals thatanalyzed in the FCS-24, between (a) the individual’s 24HR dietary intake or intake density measurement and (b) the corresponding statistical or AME disaggregated household estimate predicted for the individual based on their age group and sex. Mean bias was calculated for each food group or nutrient and within both the FCS-HH and HSES-HH by averaging bias over all 1356 individuals.
(2) Coverage probability, which was calculated as the proportion of FCS-24 dietary intake or intake density measurements contained within the 95% confidence limits of the estimate predicted by each of the two household consumption disaggregation methods that were based on each individuals’ age and sex, was assessed across all 1356 individuals analyzed in the FCS-24.
(3) For both the statistical and AME methods, disaggregated household consumption and consumption density estimates for each of the 14 age-sex groups captured by the FCS-24 sample (i.e., not including males and females aged 0–4, 5–9, and 10–14 years, which were represented in HSES-HH and FCS-HH, but not in the nested FCS-24) was assigned a rank from 1 to 14. From each rank was subtracted the rank of mean observed dietary intake or intake density for the same age-sex group in the FCS-24 to produce an age- and sex-specific absolute rank bias. Mean absolute rank bias was then calculated for each of the two disaggregation methods by averaging absolute rank bias across the 14 age-sex groups.

An additional set of the same three validation metrics were derived for each dietary component and age-sex group after applying an adjustment to the original statistical disaggregation method (hereby referred to as the “unadjusted” statistical disaggregation method and denoted as “SD1” in tables). Conceptually, the adjustment involves a hybridization of the statistical and AME disaggregation approaches to produce “AME-like” (“SD2”) estimates, by interpreting SD1′s parameter estimates not as proxies for absolute dietary intake, but instead as empirical coefficients for weighting relative consumption of observed household foods and nutrients (analogous to the AME’s method of weighting household consumption according to relative energy requirements). Equations describing the AME SD1, and SD2 methods are given in Appendix G.

### 2.9. Aim 4: Direct Prediction of Dietary Nutrient Intake by Individuals

In contrast to statistical disaggregation (prediction of household consumption to infer that of individuals), an alternate statistical approach was evaluated among the 1356 individuals that were participating in the FCS-24, in which individual dietary nutrient intakes and intake densities (adjusted for within-person variance) were directly predicted using a progressively more expansive set of household- and individual-level covariates. The purpose of this analysis was to assess—given the availability of household characteristics, individual characteristics, and individual dietary measurements for the same participants with which to train a model—the accuracy with which a household consumption and expenditure survey could predict individuals’ dietary nutrient intakes and intake densities and the relative importance of different categories of predictors. Seven such categories were evaluated in a cumulative fashion, such that the simplest model considered variables in only one category for potential inclusion, and the most complex considered five categories. Categories were added in order of increasing difficulty and invasiveness to collect, thus providing what may be considered as a more realistic hierarchy of category combinations that would be considered for assessment in an actual survey. Regularized (elastic net) regression was used to evaluate a large number of potentially significant predictors, while minimizing over-fitting (R v3.4 ‘glmnet’ package) using a natural logarithm transformation of nutrient intakes and intake densities to account for their right skew [29]. In an alternate analysis that was more comparable to that of the SD and AME disaggregation methods, nutrient intake densities were also predicted by dividing the predicted value of each nutrient intake by the predicted value of energy intake and then multiplying by 100.

For each model, shrinkage and elastic net mixing parameters λ and α were selected through nested 10-fold cross validation (the inner and outer loops of which selected λ and α values, respectively, which minimized model mean square error), using the same folds to validate λ for each value of α, as recommended by glmnet documentation. Percentage of deviance explained and mean absolute error were obtained for each model at optimal λ and α values (mean absolute error of statistical- and AME-disaggregated household estimates were also calculated for comparison). For simplicity, only in-sample fit statistics were estimated, rather than incorporating a third cross-validation loop on held-out test sets; fit statistics are therefore expected to be slightly optimistic.

## 3. Results

### 3.1. Characteristics of Study Populations

Characteristics of the FCS-HH and HSES-HH study populations are provided in Table 1. The HSES-HH oversampled households in provincial and county centers, while the FCS-HH sample more closely resembled the Mongolian population with respect to urban vs. rural locality. This difference is associated with other differences in the distributions of household size (mean: 4.0 in the FCS-HH vs. 3.3 in the HSES-HH) educational household attainment (the distribution of which was substantially narrower in the FCS-HH), family composition (single-person households were less common in the FCS-HH (7.2%) than the HSES-HH (13.1%), as was living with one’s spouse or partner (40.5% vs. 50.6%)), and the reported proportions of total household energy expenditure from impermanent members (1.81% and 2.5% in the FCS-HH and HSES-HH, respectively) and food spending outside home (12.1% and 9.1%, respectively). Mean age of household members was similar in the FCS-HH and HSES-HH samples (28.7 and 28.4 years, respectively). The use of survey weights based on urban vs. rural locality and national province are expected to compensate for some, but not all of the effect of these differences in applicable statistical analyses.

Proportions of households in the HSES-HH and FCS-HH and individuals in the nested FCS-24 observed to consume each food group and nutrient are provided in Table S3. For four nutrients in the FCS-24R, the proportion of individuals that were observed to consume them was less than 99% (alcohol (3.5%) and vitamins C (96.8%), A (96.9%), and D (93.8%)), while 20% and 11.3% of households in the FCS-HH and HSES-HH, respectively, reported any household consumption of alcohol. The proportion of individuals in the FCS-24 observed to consume any of each food group ranged from 20.1% (sugar and sweeteners) to 97.1% (meat/fish/poultry), while the proportion of observed household consumption of all food groups exceeded 90% in the FCS-HH and was less than 90% for four food groups in the HSES-HH: animal fat/eggs/dairy products (87.8%), baked and fried flour products (85.1%), starchy root vegetables (82.5%), and vegetable oils (82.5%). The correlation between consumed items was generally much higher in the FCS-HH than the FCS-24, and was higher between nutrients than between food groups in both datasets (Table S4).

### 3.2. Aim 1: Direct Comparison between Per-Capita Household Consumption and Per-Capita Dietary Measurements from the Same Households

**Figure 2:**
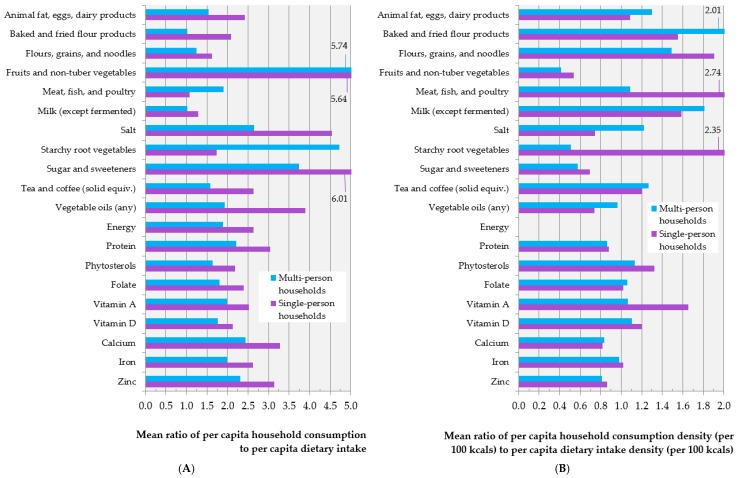
Mean ratio of per-capita household consumption to per-capita dietary intake (**A**), and per-capita household consumption density (per 100 kcal) to per-capita dietary intake density (per 100 kcal); (**B**) for food groups and selected nutrients among 109 FCS-HH households fully-enumerated by the nested FCS-24 (Aim 1). Values for 6 data points exceed the graphs’ x-axis limits and are indicated using annotations. Abbreviations: FCS-HH (2013 Food Consumption Survey), FCS-24 (nested 24-h recall).

Figure 2 and Table S1, and Table S5 compare mean per-capita household consumption and consumption density (per 100 kcal) of food groups and nutrients (derived from household recall measurements) with paired per-capita 24-h recall measurements that were collected from the same 63 multi- and 46 single-person FCS-HH households fully-enumerated by the nested FCS-24. Figure 2 summarizes the results of this analysis for food groups and selected nutrients, which may be considered to be generally more relevant to nutrition surveillance in developing countries, while Table S5 provides the results in full. Household-derived mean consumption overestimated dietary-derived mean intake of almost all food groups that were considered among members of both multi-person and single-person households, the overestimation being especially prominent among the latter. For all but two dietary components presented in Figure 2 (meat/fish/poultry and starchy root vegetables), the ratio of per-capita household- to dietary-derived means was consistently larger among single-person households. Per-capita household consumption measurements overestimated mean per-capita energy intake among multi- and single-person households by factors of 1.89 and 2.63, respectively. Across food groups, only dietary intake of baked and fried flour products, flours/grains/and noodles, and milk among multi-person households, and meat/fish/poultry and milk among single-person households were overestimated by factors smaller than 1.4, while dietary intake of sugar and sweeteners among single-person households and fruits and non-tuberous vegetables among both households types were overestimated by factors greater than 5.5. Correlation and rank correlation generally ranged from low to practically indiscernible across food groups and nutrients (correlation between per-capita energy estimates was 0.29 and 0.09 among multi- and single-person households, respectively) (Figure S1, Table S5). Only in the case of fruits and non-tuberous vegetables’ among multi-person households, and alcohol among both types of households did correlation between household- and dietary-derived per-capita estimates exceed 0.50.

With the two exceptions of phytosterols and vitamin A in multi-person households, the mean of each household-derived per-capita household consumption density (per 100 kcal) of all of the nutrients presented in Figure 2 lied within +10% of its corresponding dietary-derived mean. Conversely, food groups displayed a wide range of variation in the ratio of mean per-capita household- and dietary-derived density estimates, though generally less so than in the case of energy-unadjusted estimates, and without the same pattern of overestimation among single-person households relative to multi-person households (or overestimation overall) that was seen in the energy-unadjusted results. 

### 3.3. Aims 2 and 3: Comparison between Disaggregated Household Consumption Estimates and Individual Dietary Intake Measurements

Mean bias in the application of each household disaggregation method to the FCS-HH and HSES-HH is presented in Table 2 and is summarized graphically for food groups and selected nutrients in Figure S2. In comparison with FCS-24 dietary measurements, the unadjusted statistical method (SD1) proved more accurate than the AME method in disaggregating daily household consumption in the FCS-HH for most food groups and nutrients (mean bias in estimating individuals’ dietary energy intake for the SD1 and AME methods was +163 and +1088 kcal/day, respectively, or +8.7% and +58.0% with respect to the grand mean of dietary energy intake). Both methods tended to overestimate the consumption of most dietary components in the FCS-HH, particularly in the case of the AME method. In contrast, the application of the unadjusted statistical method to the HSES-HH produced severe underestimates of dietary energy intake (mean bias: −918 kcal/day), while the AME method still tended to overestimate, albeit to a lesser degree than in its application to the FCS-HH (mean bias: +302 kcal/day). In terms of mean bias, the statistical method performed more accurately than the AME method in estimating individuals’ dietary intake of eight of 11 food groups, but only four of 27 nutrients in the HSES-HH. In estimating dietary intake densities, the SD1 method significantly outperformed the AME method in both surveys, producing a smaller absolute mean bias for all but four or 37 dietary components in the FCS-HH and three in the HSES-HH. In disaggregation of both household surveys, the SD2 adjustments produced improvements in disaggregation intakes in the HSES-HH, but not the FCS-HH, and increased bias in intake densities in disaggregating both surveys.

Coverage probability (the proportion of FCS-24 intake measurements falling within the 95% confidence bounds of their corresponding household-disaggregated consumption estimate for the same age-sex groups) was considerably higher for the statistical methods than the AME method for disaggregating nutrient consumption in both of the surveys, with SD1 outperforming in the FCS-HH and SD2 generally outperforming in the HCES-HH (Table 3). The ability of disaggregation methods to rank dietary intake was generally poor (Table S7). Application of the SD1 method to the HSES-HH produced a smaller mean absolute bias than the AME method in assigning ranks of dietary intake across the 14 age-sex groups captured by the FCS-24 for 8 of 11 food groups, and a smaller or equal mean absolute rank bias for 15 of 27 nutrients, while SD1′s application to the FCS-HH produced a larger mean absolute rank bias for eight of 11 food groups and all nutrients. In attempting to derive ranks of mean intake density, the relative performance of the SD1 method generally improved, while the SD2 did not produce a discernible benefit to absolute bias in ranks of intake or intake density in disaggregation of either survey.

**Figure 3:**
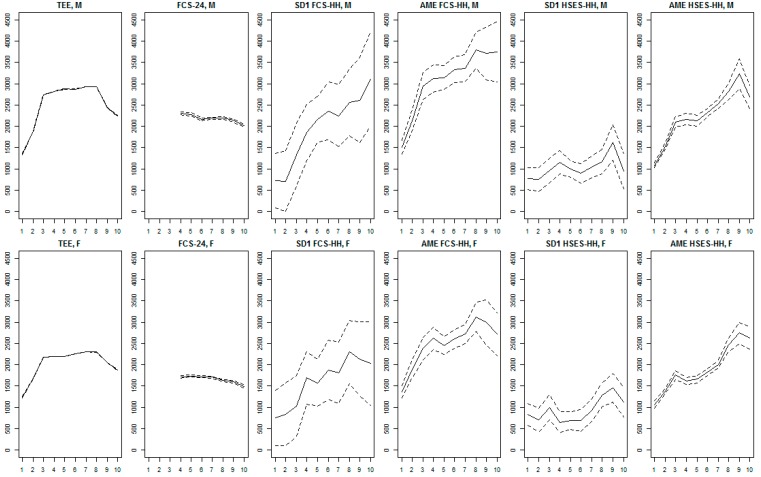
Comparison between mean daily estimated energy expenditure, observed dietary energy intake, and disaggregated household consumption estimates of individuals’ energy intake (kcal/day) across 10 age groups of males and females (Aims 2 and 3). **y-axis**: daily energy intake (kcal/day); **x-axis**: age groups 1–10 (1: 0 to 4 years, 2: 5–9, 3: 10–14, 4: 15–19, 5: 20–29, 6: 30–39, 7: 40–49, 8: 50–59, 9: 60–69, 10: 70+); **row 1**: males (“M”); **row 2**: females (“F”); **column 1**: mean predicted total energy expenditure (“TEE”); **column 2**: mean observed dietary energy intake from the FCS 24-h recall (“FCS-24”); **column 3**: unadjusted statistical disaggregation of FCS-HH (“SD1 FCS-HH”); **column 4**: adult male equivalent method (AME)-like statistical disaggregation of FCS-HH (“SD2 FCS-HH”); **column 5**: AME disaggregation of FCS-HH (“AME FCS-HH”); **column 6**: unadjusted statistical disaggregation of HSES-HH (“SD1 HSES-HH”); **column 7**: AME-like statistical disaggregation of HSES-HH (“SD2 HSES-HH”); **column 8**: AME disaggregation of HSES-HH (“AME HSES-HH”). **Solid lines** indicate means of age- and sex-specific measurements (FCS-24) or predictions (TEE and disaggregated household estimates), while **dashed lines** indicate associated 95% confidence limits. Statistics *are* survey-weighted. Abbreviations: FCS-HH (2013 Food Consumption Survey), HSES-HH (2012/2014 Household Socio-Economic Survey), AME (adult male equivalent method).

Figure 3 compares mean daily estimated energy expenditure, mean observed dietary energy intake, and disaggregated household consumption estimates of individuals’ energy intake derived from the application of the SD1, SD2, and AME disaggregation methods to the FCS-HH and HSES-HH across 10 age groups of males and females. Estimated energy expenditure among males and females exceeded the observed dietary intake in all of the age groups for which dietary intake measurements were available. Unlike estimated energy expenditure, graphs of observed dietary energy intake are relatively flat despite a slight decrease with age, while disaggregated household consumption estimates are considerably wigglier. In both the FCS-HH and HSES-HH, the standard errors confidence limits of AME-disaggregated estimates are considerably narrower than those of the statistical disaggregation estimates for the same age-sex groups.

Goodness of fit of statistical disaggregation models are provided in Table S6. FCS-HH and HSES-HH disaggregation models explained 35.0% and 34.1% of deviance in the household energy consumption, respectively (mean absolute error = 3269 and 2418 kcal/day, respectively, or 30.4% and 33.6% of the grand mean of household energy consumption). 

### 3.4. Aim 4: Direct Prediction of Dietary Nutrient Intake by Individuals

**Figure 4:**
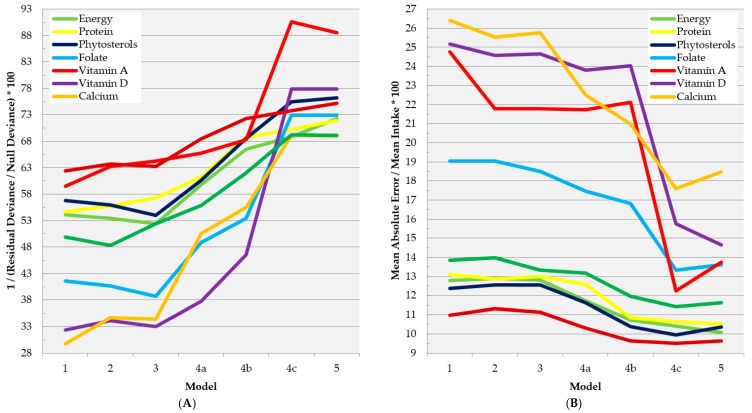
In-sample fit statistics for increasingly complex predictive models of individuals’ dietary intakes of selected nutrients in the FCS-24 (Aim 4). (**A**) percentage of deviance explained; (**B**) mean absolute bias (proportional to mean observed dietary intake). See Table 4 for detailed descriptions of models 1–5. Brief description of variable categories considered for selection in each model: (1) Household and individual demographic, socioeconomic, and lifestyle characteristics; (2) Model 1 variables + quantitative total household consumption of food groups and nutrients; (3) Model 2 variables + individuals’ self-evaluation of nutrition knowledge and its application to their lives; (4a) Model 3 variables + cursory qualitative 24-h recall and assessment of eating behaviors; (4b) Model 3 variables + cursory semiquantitative 24-h recall and assessment of eating behaviors; (4c) Model 3 variables + detailed semiquantitative 24-h recall; (5) Model 4 variables + measured anthropometry. Abbreviation: FCS-24 (nested 24-h recall of the 2013 Food Consumption Survey).

Table 4 summarizes the seven sets of variables that were considered for potential selection in each of seven increasingly complex prediction models of dietary nutrient intakes and intake densities by individuals in the FCS-24. Detailed in-sample fit statistics for these models are presented in Table S8 and S9, and graphically in Figure 4 (intakes) and S3 (intake densities), for a subset of nutrients that are generally more relevant to surveillance in developing countries. The most basic model (Model 1), incorporating only household and individual demographic, socioeconomic, and lifestyle variables, explained 53.6% of daily caloric intake with a mean absolute bias of 229 kcal/day (as compared with 384 and 1095 kcal/day from SD1 and AME disaggregation of the FCS-HH, respectively) (Figure 4, Table S8). Increasing model complexity by adding household food group and nutrient intake (Model 2) and/or individual nutrition knowledge (Model 3) to the pool of selectable variables produced modest increases in the predictive ability (>+4% deviance explained) for total fat, certain vitamins (riboflavin, vitamin B12, vitamin C, and vitamin A), and calcium, while the effects on other macronutrient and micronutrients prediction were smaller (Figure 4, Table S8). The marginal benefit of including measured anthropometry (Model 5) was generally small to negligible. Occasionally, the addition of these variables (household consumption, nutrition knowledge, measured anthropometry) to the pool of potential predictors appeared to “confuse” model selection and result in a slightly poorer model fit.

The largest and the most consistent marginal improvements to model fit were incurred upon inclusion of cursory qualitative, cursory semiquantitative, or detailed semiquantitative assessment of diet and eating behaviors (Models 4a–4c), particularly for micronutrients. For example, in the case of calcium, the addition of cursory qualitative, cursory semiquantitative, or detailed semiquantitative diet variables to model selection resulted in a 16.2%, 4.9%, and 13.7% increase in deviance explained, respectively, and an overall 34.8% net decrease in mean absolute error relative to the grand mean of calcium intake in the FCS-24 (Figure 4, Table S8). In the majority of cases, the accuracy with which the same model predicted dietary intake densities for a given nutrient (in terms of deviance explained) was poorer than that of predicting intakes, especially prior to the addition of detailed semiquantitative diet variables. Nonetheless, for all nutrients, mean absolute error of even the simplest intake density prediction models (Model 1) was less than those of the statistical and AME disaggregation methods. Results were similar for alternate prediction nutrient density prediction models that were based on the separate prediction of nutrient intake and energy intake (Figure S3 and Table S9). 

Note: The number of significant figures reported in estimates of and bias in dietary intake and household consumption of nutrients reflect the precision of laboratory analytical measurements of nutrient concentrations, while the number of significant figures in estimates of and bias in nutrient intake and consumption densities (per 100 kcal) is deliberately increased by 1 for the ease of interpretation. Statistics are not survey-weighted unless indicated otherwise.

## 4. Discussion

Given the dearth of detailed, periodic dietary intake data for much of the world’s population and the volume of food consumption data that is present in household consumption and expenditure (HCE) surveys, the potential value of HCE data to nutrition research and surveillance is immense, particularly for developing countries. In recognition of this, recent decades have seen steadily growing interest in survey design and analytical approaches geared toward increasing the applicability of HCE data in nutrition [7,30,31]. This effort is challenged by the fact that household food and nutrient consumption are far from perfect proxies for individuals’ diets, the primary exposure of interest in nutritional epidemiology and one that remains difficult to assess with great accuracy even under the best of conditions. Nonetheless, necessity is the mother of invention, and some interesting ways to meet this challenge have suggested themselves in the literature, four of which are evaluated in this paper.

### 4.1. Aim 1: Direct Comparison between Per-Capita Household Consumption and Per-Capita Dietary Measurements from the Same Households

The first and simplest approach involves direct inference of dietary intake based on per-capita household food consumption [32,33,34,35]. Accurate household consumption measurements are a prerequisite for accuracy of the AME and statistical disaggregation methods that are evaluated in this paper; for the purpose of directly assigning dietary intake to individuals, per-capita household consumption measurements are less useful for multi-person households because they imply impossibly equitable intra-household distribution of food. Because persons living by themselves are the main consumers of food in their household (with the exception of guest and visitors), household food consumption may be an appropriate proxy for these individuals’ dietary intakes, although the degree to which these estimates are generalizable to those living in multi-person households may be limited. In the current study, we found household food group and nutrient consumption among 109 FCS-HH households fully-enumerated by the FCS-24 to overestimate and correlate poorly with dietary intake in both types of households, especially for single-person households. 

Important sources of systematic and random error are known to influence reporting of household food consumption data [10] and have more recently been subject to more formal decomposition [36]. In particular, the magnitude of overestimation in the FCS-HH suggests that enumerators provided telescoped estimates (their recall included household foods consumed prior to the reference period) [36,37]. It is also plausible that reported household food consumption was partly conflated with food that was acquired (or simply present in household stocks) over the reference period, but that not necessarily consumed, or was perhaps transferred to other households. It is not immediately clear why over-reporting would affect multi-person households to a lesser extent than single-person households, but this may have to do with accuracy that is incurred by the cognitive exercise of distinguishing and dividing consumption among each household member in a multi-person household, while those living alone might rely on less enumerative rules-of-thumb. Recall error is mitigated by the use of prospective instruments such as the HSES-HH’s consumption diary, but these are conversely more likely to be affected by underreporting due to respondent burden [21]. Both retrospective and prospective assessment methods are also subject to some measure of social desirability bias (the latter by way of respondent “self-monitoring”) [38], however, research is lacking on the importance of this bias in assessing diet and household food consumption in Mongolia. Efforts to improve the accuracy of household food consumption measurements are ongoing and have considered such cognitive and survey design [7,39]. In Mongolia, recent analysis by Troubat and colleagues suggests that the HSES-HH’s diary instrument could be satisfactorily substituted with a less costly consumption recall combined with measurement of changes in household foods stocks and acquisitions [21].

Per-capita estimates of household consumption density more closely agreed with dietary-derived per-capita intake densities in both household types, particularly in the case of nutrients. To some degree, this agreement may be inflated by shared systematic error in the reporting of foods in the FCS-HH and FCS-24 (e.g., the fact that both rely on memory and self-report), as well as nutritional analysis (e.g., the fact that the same food composition data was used to analyze both datasets) [40]. The observed agreement is nonetheless encouraging given that nutrient densities are meaningful nutrition indicators in and of themselves, and which provide a convenient way to compare individuals with different caloric intakes despite the aforementioned sources of error (both of which are non-differential) [28].

An important area of innovation in dietary and household surveys globally is the emerging use of technology-assisted assessment of food consumption, as well as commercially-available retail and procurement data from supermarket sales and purchases [41,42,43]. To our knowledge, technology-assisted collection methods are not widely used in Mongolian national surveys, including in urban areas. Since 2016, the Mongolian Tax Administration has collected extensive information about individuals’ purchases of specific goods and services in Mongolia using an advanced electronic database system [44,45] for the purpose of issuing tax refunds, however, it is not clear how applicable this information may be to the analysis of food consumption patterns.

### 4.2. Aims 2 and 3: Comparison between Disaggregated Household Consumption Estimates and Individual Dietary Intake Measurements

Next, we evaluated the validity of the AME method to disaggregate household food and nutrient consumption based on household members’ relative caloric requirements. The validity of this method has been evaluated in numerous surveys outside Mongolia. In studies of two household consumption and expenditure surveys in Uganda, the AME method provided reasonable estimates of dietary nutrient density, but more often underestimated the dietary intake of potential fortification vehicles among women and children in comparison with results of a nested 24-h recall, varyingly explained by the inability of each survey’s household instrument to fully enumerate-foods consumed and the extent to which the intra-household distribution of staple foods in Uganda is disproportionate to the caloric requirements of household members [46,47]. By contrast, an analysis of 4195 Bangladeshi households revealed the AME method to produce remarkably accurate disaggregated estimates of most nutrients’ dietary intake in comparison with the results of 24-h recalls collected from the same study population, implying that the consumption of most foods would likely be accurately disaggregated as well [48]. This finding was corroborated by a pooled analysis of six Bangladeshi surveys including 1232 households, which found that in Bangladesh, more so than in most of the 13 other countries for whom similar pooled analyses were undertaken, intra-household distribution of consumed calories appears to be relatively proportional to intra-household distribution of caloric requirements (this is a necessary, though not sufficient prerequisite for intra-household distribution of foods and non-caloric nutrients in a manner that is proportional to caloric requirements, which is a cardinal assumption of the AME method) [16].

In the present study, the application of the AME method to the larger HSES-HH showed it to be generally more apt than the statistical method at estimating and ranking individuals’ intakes of dietary components, but it also overestimated intake in both household surveys and produced extremely narrow standard errors. The latter may be attributed to the AME method’s relatively deterministic manner of disaggregating consumption, which could be addressed by assigning more granular estimates of energy expenditure (or by deliberately assigning error to estimates, drawn from error observed in energy expenditure prediction models [49]). On the other hand, a benefit of a deterministic approach is that it does not imply a sample size requirement to produce precise disaggregated estimates (unlike the statistical method). With regard to the comparative accuracy of the AME method, some investigators specifically suggest that its strength lies in estimating the intake of those dietary components more correlated with energy [50,51]. Accordingly, in disaggregation of the HSES-HH, the AME method more accurately estimated individuals’ intakes of animal fat/eggs/dairy products, baked and fried flour products, and flours/grains/noodles, which are the major staples of the Mongolian diet, and which are relatively calorie-dense and nutrient sparse. To the extent that dietary intake of caloric energy, macronutrients, and staple foods (for example, fortifiable flour) are ubiquitous and are subject to homeostatic regulation [52], predicting individuals’ intakes of these dietary components should require a disaggregation method to be less discriminating of components of variation in intra-household food consumption, which are attributable to prevailing social or cultural forces rather than biological ones. In such cases, it may be more reasonable to depend on the AME method than the statistical method, the latter of which may incur statistical error without a discernible benefit to accuracy. The AME method may also be extended to a more generalized concept of intra-household “equivalency scales” by weighting nutrient household consumption according to nutrient requirements other than that of energy [50]. If household food consumption is reported inaccurately, however (as verified in the case of the FCS), the AME method will produce biased estimates regardless of dietary components’ known associations with energy or other nutrients’ intake or requirements.

Unlike the AME disaggregation method, the statistical method has not been previously validated. The plausibility of dietary intake estimates that are produced by the statistical method generally supported in the literature by its apparent ability to predict natural variation in caloric intake with age—increasing intake during childhood, a spike in puberty, and a decrease later in life—rather than by comparison with actual consumption data for energy or other dietary components (which had not previously been studied) [18,27,53,54,55,56,57,58,59]. Despite this, an advantage of a more data-driven statistical method over that of the AME would be expected in the case of dietary components, whose consumption is less correlated with energy requirements (e.g., most foods (Table S4)), and those that are less correlated by definition (all food group and nutrient intake densities). Accordingly, the statistical method more accurately assigned dietary intake of food groups and intake densities of both food groups and nutrients in disaggregating both household surveys.

An interesting aspect of the statistical method is that its inclusion of a model intercept accommodates the possibility that not all household food is consumed, and thus ought to be disaggregated, which could be important if household consumption were measured in terms of proxies, such as food expenditure, acquisitions, or stocks; this is suggested by Chesher in the method’s initial application to food acquisitions among British households [18]. In disaggregating surveys, which explicitly measure household food consumption (such as those analyzed in this study), the intercept explicitly represents consumption that is unrelated to the number, age, or sex of individuals living in each household, which may be useful if it helps account for food that was reported to be consumed, but which was in fact merely acquired, present in the house, but not consumed, given to animals, wasted, or which spoiled. This usefulness is supported by the statistical method’s comparative accuracy in disaggregating consumption in the FCS-HH (Aim 2) despite this survey’s overestimation of per-capita dietary intake (Aim 1). The utility of the intercept in this regard requires that household consumption is over-reported in an additive rather than a multiplicative fashion, otherwise the differences between predicted intakes across age-sex groups will be inflated (as will the model intercept); we have affirmed this experimentally by applying the statistical method after adjusting household consumption using either a constant or a multiplier (not shown). Conversely, to the extent that household food consumption is multiplicatively underestimated, the intercept will be attenuated, as will the differences in predicted intake across age-sex groups. This may have been responsible for the statistical method’s poor performance in the disaggregation of the HSES-HH (which likely experienced multiplicative underreporting associated with the burden of the diary instrument), and why performance improved after removing the model intercept in applying the AME-like (“SD2”) adjustment. Thus, while the statistical method depends less on assumptions of accurate reporting of household consumption per se than the AME method, it is nonetheless influenced by the nature of this inaccuracy. 

The statistical method is potentially limited in ways that the AME method is not, stemming from its reliance on accurate and precise prediction of household food consumption (without which accurate or precise estimates of dietary intake among different age-sex groups may not be inferred). For example, zero-inflation in the distribution of household food consumption due to the presence of non-consumers over the reference period may produce poor model fit and inaccurate predictions (Table S2). In this study, our use of zero-inflated models implies that non-consuming households would in fact be consumers given a longer reference period, which is likely a reasonable assumption for most food groups and nutrients (except alcohol), but that may not be reasonable were smaller (less aggregated) food groups to be analyzed. In such cases, a two-part or “hurdle” model which deliberately distinguishes between processes of household consumption frequency and consumption magnitude may be more appropriate for modeling mean household consumption in the population. With regard to precision, while smoothing parameter estimates may be helpful for producing more realistic estimates, the degree of smoothing is a subjective choice that may obscure rather than expose true variation in predicted dietary intake with age, particularly if the imprecision in estimates is severe. In this study, model fit of statistical disaggregation models was generally poor (Table S6). Improving precision is challenged by the fact the inclusion of highly predictive variables—household energy intake or household size—e.g., changes the interpretation of parameter estimates, such that they reflect effects on household composition rather than the addition of household members (partly defeating the purpose of using the statistical method over the AME method, the latter of which is necessarily dependent upon assumptions of intra-household distribution).

### 4.3. Aim 4: Direct Prediction of Dietary Nutrient Intake by Individuals

Finally, we attempted to estimate individuals’ dietary intakes and intake densities using a prediction model that was incorporating household food consumption and other data feasibly obtainable from a household survey, with relatively precise results. While examples of this approach are relatively sparse in the literature [51], we derived what we consider to be acceptably precise predictions of dietary nutrient intake and intake densities. Given the predictors that are available for model selection, the results were similar between models directly predicting nutrient intake densities vs. those based on separate prediction of nutrient and energy intake. Similar to the statistical disaggregation method, the prediction model does not require potentially inaccurate assumptions about the intra-household distribution of food consumption. Prediction further relaxes assumptions that the reporting of household food consumption is systematically (as in the case of the AME method) or differentially (as in the case of the statistical method) unbiased with respect to dietary intakes of household members, and offers more flexibility with respect to potential effect modifiers or confounders. For example, we found in Aim 1 that bias in per-capita household consumption was differentially affected by household size. In Aims 2 and 3, we found that despite attempting to control for household educational attainment, family composition, outside food spending, consumption by impermanent members, and locality, a strong pattern of increasing estimated intake in advanced age was observed in both the AME and statistical disaggregation estimates for most foods and nutrients, which is contrary to what we expected based on both dietary energy intake and predicted energy expenditure. This pattern may result from residual confounding by socioeconomic status and household size, in that wealthier Mongolian households generally consume more food, are smaller (increasing per-capita food consumption), and their members have longer life expectancies [60]; it is also possible that smaller (and younger) households underreported food consumption, according to the cognitive hypothesis that was discussed previously in Aim 1 (to some extent, increasing intake with age may also be real, given that the Mongolian population is still relatively young and older individuals are more metabolically active than their counterparts in other populations). In addition to more efficient control for confounding variables, prediction allows for estimates to be produced across more granular strata of individuals, while the statistical method may only do so with difficulty (for example, by analyzing strata independently and reducing statistical power, or by introducing a potentially unwieldy number of interaction terms between age-sex groups and covariates of interest [18]). 

Based on our prediction models, we suggest that household surveys would be well adapted to estimate dietary intake and intake densities by the addition of a rudimentary dietary assessment module. Predictive approaches have performed well in analysis of food frequency questionnaires (FFQs), the rationale being that such an approach acknowledges “the importance of a food item should reflect not only the nutrient content of the food, but also the validity of the responses to that particular item” [37]. Existing platforms for conducting household surveys would be well-suited for applying this method, given that they are prepared using large, nationally-representative sample frames, and are collected periodically. The expense of a validation study (i.e., simultaneous collection of dietary intake data with which to build a model) should not be considered as a limiting factor, as it will also produce useful consumption estimates that could otherwise have been collected in a separate dietary survey; even cursory qualitative information about individuals’ diets can be useful for assessing food security or screening for chronic disease risk [6,61]. Still, some may question the purpose of adding dietary assessment of individuals to a household survey in lieu of conducting a more rigorous standalone dietary assessment. If resources were available to do so, then measurements that are collected in such a survey would assuredly be more accurate than those that were obtained through prediction. If resources are not available, however, prediction may offer a reasonable compromise between an infeasible approach and no approach at all. Furthermore, while it is not unreasonable to append a qualitative or semiquantitative dietary assessment module to an HSES questionnaire, more involved dietary measurements (such as diet records or a 24HR) may diminish compliance and compromise accurate collection of other survey modules. For the purpose of prediction, the level of detail at which to collect individuals’ dietary and eating behavior information—cursory qualitative, cursory semiquantitative, or detailed semiquantitative—should be carefully considered in the context of a given HCE platform, not all of which may be suited to accommodate a highly detailed questionnaire. This should not preclude the consideration of more detailed quantitative or semiquantitative food frequency questionnaires, however (the value of which could not be evaluated in this study given the use of the 24HR). If an FFQ were used, a predictive model framework in the context of HCE data may enhance the instrument’s usefulness in collecting absolute intake, while in the case of a 24HR, it may increase the instrument’s ability to assess long-term diet.

Finally, household consumption data and household characteristics may also provide added value when being collected as part of a dietary survey or otherwise in the context of national dietary data (rather than simply as a less expensive alternative to dietary data) by supplying complimentary information on the intra-household distribution of food consumption and nutrient adequacy, and household-level predictors of food security and dietary diversity [5,6,12], which would not generally be assessed in a dietary survey. Furthermore, while dietary surveys are collected using a variety of methodologies, efforts to standardize household survey instruments have rendered household consumption data generally more appropriate for international comparisons, and these data may therefore be generally more applicable to multilateral policymaking [30].

### 4.4. Strengths and Limitations

By disaggregating two household surveys from the same national population using two different instruments for assessing household food consumption (a recall and a diary), this study was able to assess the reproducibility of disaggregated household consumption estimates and study differences in the survey design. The size of the HSES-HH allowed for more statistically powerful disaggregation, while the FCS-HH, although a smaller survey, was conducted in the same population as the dietary assessment, and thus allowed for an inherently more direct and multi-faceted comparison. Comparability of the two household surveys was strengthened in that both were nationally-representative, seasonally-matched, and conducted within two years of one another. Analysis of both individual dietary intake and household food consumption incorporated local and empirical food yield, food composition, and physical activity, and incorporated empirical estimates of food eaten outside of the home, allowing for more a more rigorous validation.

An important limitation of this study is potential underreporting by the 24-h recall. The extent to which this has affected the comparative validity of the AME and statistical disaggregation methods is expectedly mitigated inasmuch as this underreporting affected both household surveys in a similar fashion, the fact that all of the disaggregated household results were compared to the same dietary assessment, the fact that dietary underreporting should not necessarily be expected to differentially bias reported or predicted intake of a given food group or nutrient across different age and sex groups; in validating both per-capita estimates (Aim 1) and disaggregated estimates (Aims 2 and 3), underreporting was further mitigated by conducting energy-adjusted analysis [28]. Second, while being suitable for assessing mean dietary intake, a single 24HR does not provide estimates of usual intake. Adjustment for within-person variance using variance components from the same national population helped to account for this limitation in the case of nutrients, but not food groups. Third, while various factors were applied to render household food consumption measurements comparable with dietary intake, including consumption by impermanent household members, we were unable to account for guests and visitors who affect household food supplies, but were not accounted for by the surveys that were analyzed. A final limitation of this study was the lack of information on individual dietary intake by children, or by any age groups in seasons other than summer, making it impossible to determine the validity of the method for children or in different seasons in Mongolia. Further research is warranted to address this.

## 5. Conclusions

In this study, we found per-capita household consumption to be an adequately accurate measure of per-capita nutrient intake density, the statistical disaggregation method to be generally less biased than the AME method in estimating individuals’ intakes of dietary components that were less correlated with energy (while the converse was sometimes true for the AME method), and the direct prediction of individuals’ intakes and intake densities—while subject to the requirement of available dietary data with which to build a prediction model—to be the most precise method (particularly when it incorporated basic information on household members’ diets and eating behaviors). We note that each of different estimation methods has its own strengths, weaknesses, and applications, and that their performance depends importantly on survey-specific factors that may vary widely both between and within countries. In light of these observations, we find it inappropriate to categorically recommend one method over another, or to recommend against estimation entirely to focus more on measuring diet directly (notwithstanding the importance of ongoing efforts to advance dietary data collection globally, which should continue to be supported). We also support continuing efforts to capture household food consumption more accurately. In order to render these data more useful for applications in nutrition, they should be collected in ways that facilitate accurate disaggregation [4,8,62], including the collection of ancillary data on intra-household food distribution or diet from at least a subset of survey households (the latter of which is compatible with our recommendations regarding expanded use of prediction models). We suggest that it would be valuable to conduct similar prediction exercises in other countries, evaluating different types of household and individual assessment instruments.

## Figures and Tables

**Table 1:** Characteristics of households and individuals in the FCS-HH and HSES-HH.

	Characteristic	FCS-HH	HSES-HH
**Household Characteristics**	Households (*n*)	1012	9424
	Location, *n* (%)		
	Ulaanbaatar	472 (46.6)	2332 (24.7)
	Provincial/county center	168 (16.6)	4937 (52.4)
	Rural	372 (36.8)	2155 (23.3)
	Household size, mean (SD)	4.0 ± 1.7	3.3 ± 1.6
	Family composition *n* (%)		
	1 man	40 (4.0)	574 (6.1)
	1 woman	34 (3.4)	662 (7.0)
	2 or more adults	326 (32.2)	2922 (31.0)
	Adult(s) and children	612 (60.5)	5235 (55.5)
	Children only	0 (0.0)	31 (0.3)
	Maximum education (years), *n* (%)		
	0 to 4	32 (3.2)	694 (7.4)
	6 to 10	593 (58.6)	4719 (50.1)
	14+	387 (38.2)	4011 (42.6)
	% TEE from impermanent members, mean (SD)	1.81 ± 3.28	2.5 ± 8.2
	% food spending outside home, mean (SD)	12.1 ± 12.3	9.1 ± 24.6
	Household TEI/TEE, mean (SD)	1.35 ± 0.65	1.09 ± 0.79
**Individual Characteristics**	Individuals (*n*)	4070	34,946
	Age (years), mean (SD)	28.7 ± 19.6	28.4 ± 19.1
	Sex, *n* (%)		
	Female	2140 (52.6)	17,873 (51.1)
	Male	1930 (47.4)	17,073 (48.9)
	Married or living with partner, *n* (%)	1648 (40.5)	17,667 (50.6)
	TEI/TEE, mean (SD)	0.77 ± 0.14	-

Values indicate *n*, *n* (%), or mean ± SD. Statistics are derived after restricting HSES-HH data to those collected in May, June, July, or August, and after applying exclusion criteria. % household total energy expenditure (TEE) from guests and visitors and % of food spending are expressed as “during the reference period” of each survey. Four years of education corresponds to completion of primary school; 6: currently in secondary school; 10: completed high school or vocational training; 14: completed bachelor degree. Abbreviations: FCS-HH (2013 Food Consumption Survey), HSES-HH (2012/2014 Household Socio-Economic Survey), TEI (total energy intake).

**Table 2:** Mean bias of disaggregated household consumption estimates of individuals’ food group and nutrient intake and intake density (per 100 kcal) across 14 age-sex groups (Aims 2 and 3).

Validation Metric:		Mean Bias in Intake							Mean Bias in Intake Density (Per 100 kcal)						
Household Survey:		FCS-HH (*n* = 1012)				HSES-HH (*n* = 9424)			FCS-HH (*n* = 1012)				HSES-HH (*n* = 9424)		
Disaggregation Method:		Intake	SD1	SD2	AME	SD1	SD2	AME	Density	SD1	SD2	AME	SD1	SD2	AME
**Food Groups**	Animal fat, eggs, and dairy products (g)	92.1	−6.3	109.7	128.6	−61.9	−13.2	16.2	4.90	−0.72	1.19	5.44	−1.32	−1.16	3.41
	Baked and fried flour products (g)	115.0	−16.2	64.1	63.6	−75.0	0.3	19.6	6.06	−1.23	−0.54	2.22	−1.62	−0.57	4.09
	Flours, grains, and noodles (g)	231.9	−29.7	49.0	64.1	−146.8	−48.6	−38.8	12.06	−1.75	−2.99	1.17	−2.96	−3.29	−0.17
	Fruits and non-tuber vegetables (g)	31.6	9.1	101.5	90.0	−7.7	42.7	57.2	1.77	0.27	2.57	5.09	0.63	1.94	5.17
	Meat, fish, and poultry (g)	114.4	2.7	126.7	89.0	−47.6	50.7	53.4	5.88	−0.14	1.67	2.77	0.99	1.87	3.97
	Milk (except fermented) (g)	77.9	36.3	232.6	189.6	0.4	133.2	148.7	4.18	1.38	5.33	8.21	3.94	5.23	9.69
	Salt (g)	1.8	2.8	6.7	6.0	−0.3	3.2	3.5	0.10	0.14	0.17	0.38	0.07	0.14	0.34
	Starchy root vegetables (g)	30.7	35.4	75.7	82.4	−0.8	28.3	42.9	1.69	1.61	1.70	5.02	1.44	1.15	4.19
	Sugar and sweeteners (g)	3.6	7.6	14.1	16.9	4.3	13.7	14.8	0.20	0.35	0.35	0.79	0.63	0.61	1.18
	Tea or coffee (solid equivalent) (g)	3.6	−1.0	1.8	1.8	−1.1	2.4	2.2	0.20	−0.07	−0.02	0.13	0.03	0.08	0.31
	Vegetable oils (any) (g)	6.6	1.3	8.7	8.9	−2.5	2.7	5.4	0.33	0.05	0.14	0.43	0.09	0.10	0.69
**Macronutrients**	Energy (kcal)	1864	163	1335	1088	−918	267	302	N/A	N/A	N/A	N/A	N/A	N/A	N/A
	Carbohydrates (g)	241.10	19.13	132.57	123.41	−127.89	4.79	16.54	12.920	0.052	−1.166	0.890	−0.783	−1.295	0.669
	Protein (g)	70.09	7.54	64.14	49.69	−32.71	18.11	18.35	3.777	0.032	0.377	0.319	0.192	0.362	0.637
	Total fat (g)	66.38	3.62	67.62	47.04	−29.79	20.24	21.98	3.574	−0.194	0.523	0.220	0.235	0.471	0.268
	Alcohol (g)	1.47	−1.23	−0.90	−0.51	−1.46	−0.88	−0.18	0.067	−0.049	−0.055	0.204	−0.048	−0.040	0.441
	Water (g)	572.27	96.92	704.75	558.11	−208.99	335.68	325.36	31.081	1.629	8.337	11.702	6.622	11.052	18.846
	Fiber (g)	8.6	1.6	6.4	5.8	−4.0	0.8	1.4	0.47	0.04	0.01	0.11	0.03	−0.01	0.10
	Phytosterols (mg)	424	104	309	256	−229	−22	−9	22.9	3.6	0.2	3.7	−1.9	−3.8	0.5
**Vitamins**	Thiamin (mg)	0.784	0.164	0.723	0.563	−0.318	0.228	0.278	0.0426	0.0043	0.0045	0.0073	0.0071	0.0054	0.0148
	Riboflavin (mg)	1.220	0.184	1.225	0.990	−0.486	0.463	0.484	0.0661	0.0033	0.0103	0.0127	0.0097	0.0119	0.0216
	Niacin (mg)	13.064	2.539	12.945	9.419	−5.335	3.891	4.392	0.7093	0.0625	0.1066	0.1304	0.1044	0.0910	0.2470
	Pantothenic acid (mg)	3.111	0.668	3.099	2.383	−1.238	1.218	1.146	0.1686	0.0176	0.0247	0.0240	0.0265	0.0336	0.0406
	Vitamin B6 (mg)	0.628	0.150	0.684	0.534	−0.279	0.170	0.187	0.0342	0.0044	0.0071	0.0124	0.0022	0.0036	0.0119
	Folate (µg)	132	4	95	76	−65	21	27	7.1	−0.4	0.0	0.2	0.2	0.1	1.2
	Vitamin B12 (µg)	6.35	−0.85	2.29	1.98	−3.00	0.48	0.54	0.339	−0.058	−0.057	0.019	−0.007	−0.021	0.036
	Vitamin C (mg)	12.4	4.1	24.0	20.7	−3.4	10.0	12.6	0.70	0.12	0.45	1.00	0.26	0.39	0.99
	Vitamin A (µg)	448	−112	187	173	−200	−2	54	23.7	−6.6	−2.8	6.5	0.6	−2.5	4.7
	Vitamin D (IU)	26	1	30	22	−12	10	12	1.4	−0.1	0.3	0.6	0.0	0.3	0.4
	Vitamin E (mg)	5.28	0.24	5.24	4.33	−2.68	0.92	1.57	0.286	−0.016	0.040	0.113	−0.008	0.010	0.137
**Minerals**	Calcium (mg)	432	100	544	466	−151	255	288	23.6	2.4	6.4	9.2	5.8	8.1	13.6
	Copper (mg)	0.986	0.097	0.600	0.483	−0.447	0.100	0.119	0.0528	0.0019	−0.0019	0.0065	0.0035	−0.0010	0.0078
	Iron (mg)	10.03	1.07	7.47	5.84	−4.73	1.53	1.92	0.541	0.009	0.007	0.044	0.027	0.006	0.102
	Magnesium (mg)	168	29	141	115	−77	41	41	9.1	0.7	0.6	1.0	0.6	0.8	1.6
	Manganese (mg)	2.172	0.196	1.308	1.220	−0.998	0.394	0.434	0.1171	0.0008	−0.0075	0.0193	0.0073	0.0040	0.0347
	Phosphorus (mg)	907	93	835	660	−446	200	211	48.9	0.4	5.1	3.5	0.4	2.8	5.8
	Potassium (mg)	1436	207	1637	1209	−620	625	591	78.1	2.1	16.7	18.0	7.7	18.7	27.5
	Zinc (mg)	10.85	0.91	11.20	7.80	−4.97	3.58	3.52	0.587	−0.011	0.096	0.084	0.030	0.086	0.159

Mean dietary intake and intake density estimates from the FCS-24 are provided for better interpretability of mean bias in disaggregated estimates. Green-Yellow-Red shading indicates the magnitude of absolute mean bias in estimated intake in proportion to mean intake (Green: minimum observed absolute mean bias; yellow: median; red: maximum), and Blue-Yellow-Red shading indicates the magnitude of absolute mean bias in estimated intake density in proportion to mean intake density (Blue: minimum observed absolute mean bias; yellow: median; red: maximum). Statistics are survey weighted. Abbreviations: FCS-HH (2013 Food Consumption Survey), FCS-24 (nested 24-h recall), HSES-HH (2012/2014 Household Socio-Economic Survey), SD1 (unadjusted statistical disaggregation method), SD2 (AME-like statistical disaggregation method), AME (adult male equivalent method), IU (international unit; 40 IU = 1 μg).

**Table 3:** Coverage probability of household disaggregation methods across 14 age-sex groups (mean % of observed dietary nutrient intakes or intake densities (per 100 kcal) lying within 95% confidence interval of corresponding disaggregated household consumption estimate) (Aims 2 and 3).

Validation Metric:		Nutrient Intake Coverage Probability				Nutrient Density Coverage Probability			
Household Survey:		FCS-HH (*n* = 1012)		HSES-HH (*n* = 9294)		FCS-HH (*n* = 1012)		HSES-HH (*n* = 9294)	
Disaggregation Method:		SD1	SD2	AME	SD1	SD2	AME	AME	AME
**Macronutrients**	Energy (kcal)	88.5	13.2	3.0	10.4	37.5	13.8	N/A	N/A
	Carbohydrates (g)	76.7	25.8	5.2	3.2	23.2	10.7	37.0	17.8
	Protein (g)	79.9	18.0	2.0	8.1	24.1	8.1	24.2	2.8
	Total fat (g)	79.1	26.4	5.3	33.1	46.4	16.1	27.9	8.6
	Alcohol (g)	45.4	26.4	27.5	37.8	32.2	7.4	1.6	0.1
	Water (g)	71.5	20.3	3.9	21.8	10.8	3.2	8.9	2.4
	Fiber (g)	76.3	12.4	2.0	6.8	32.6	11.4	13.4	6.0
	Phytosterols (mg)	72.5	18.9	2.7	3.4	23.2	16.3	22.0	29.3
**Vitamins**	Thiamin (mg)	71.0	13.5	2.5	16.0	28.7	7.0	17.4	1.6
	Riboflavin (mg)	79.5	23.4	2.7	16.8	21.2	5.0	14.8	2.0
	Niacin (mg)	75.9	7.0	1.6	10.5	25.2	5.1	21.4	1.4
	Pantothenic acid (mg)	71.3	14.0	2.5	15.5	17.4	4.7	19.1	2.4
	Vitamin B6 (mg)	71.0	10.7	2.7	13.0	20.8	5.4	10.5	3.5
	Folate (µg)	80.8	24.3	6.7	9.1	23.7	8.1	24.6	5.5
	Vitamin B12 (µg)	71.6	40.7	12.0	20.9	23.9	15.0	23.8	7.1
	Vitamin C (mg)	84.6	14.9	0.6	50.3	9.6	0.7	1.6	0.1
	Vitamin A (µg)	69.3	30.8	12.3	59.0	49.4	13.6	9.7	3.0
	Vitamin D (IU)	76.2	28.6	4.5	67.0	47.1	13.4	13.8	7.9
	Vitamin E (mg)	81.9	24.3	3.6	11.1	38.2	11.1	11.5	2.7
**Minerals**	Calcium (mg)	80.2	25.7	5.1	30.5	17.2	4.4	10.7	1.8
	Copper (mg)	63.8	21.0	7.6	12.5	26.4	10.1	18.0	6.2
	Iron (mg)	77.2	15.1	3.3	6.8	27.3	8.2	31.0	2.7
	Magnesium (mg)	68.4	14.2	2.4	11.5	22.9	9.4	17.4	2.7
	Manganese (mg)	78.4	19.7	2.9	8.1	27.0	9.7	24.8	2.2
	Phosphorus (mg)	79.7	16.9	2.7	9.6	23.9	9.9	25.3	5.6
	Potassium (mg)	74.4	15.4	1.8	15.4	14.6	4.1	10.4	0.8
	Zinc (mg)	82.3	16.6	1.9	7.9	26.2	7.3	21.8	1.9

Shading indicates the magnitude of estimated mean coverage probability (Green: maximum estimated coverage probability; yellow: median; red: minimum). Mean coverage probability is omitted for statistical (SD1) and AME-like (SD2) nutrient intake densities given the complexity of deriving standard errors for the corresponding ratio estimators. Abbreviations: Abbreviations: FCS-HH (2013 Food Consumption Survey), HSES-HH (2012/2014 Household Socio-Economic Survey), SD1 (unadjusted statistical disaggregation method), SD2 (AME-like statistical disaggregation method), AME (adult male equivalent method), IU (international unit; 40 IU = 1 μg). Statistics are survey weighted.

**Table 4:** Categories of household- and individual-level variables considered for selection in predictive models of individuals’ dietary nutrient intakes and intake densities in the FCS-24 (Aim 4).

		Models in Which Each Category Was Considered for Selection							
Category	Variables Comprised by Each Category	1	2	3	4a	4b	4c	5	
Household and individual demographic, socioeconomic, and lifestyle characteristics	Household-level variables: Weekday of assessment; province and location (capital, provincial/county center, rural) of household; numbers of men, women, boy, and girl household members; presence of students, herders, pensioners, married men or women, and members of the agricultural, industrial, or service industries in the household; total household income; average daily value of all foods consumed by the household; average daily value of foods eaten outside home; sum and maximum of household members’ years of education; household family composition; average daily energy expenditure of all household members; average daily energy expenditure of all guests and visitors. Individual-level variables: Age, sex, relationship to head of household, marriage status, current pregnancy or lactation, years of education, occupation, industry of employment, any food allergy, self-evaluated physical activity level; overall health, presence of any metabolic disease, and presence of any other serious disease in past 6 months.	**√**	**√**	**√**	**√**	**√**	**√**	**√**	
Quantitative total household consumption of food groups and nutrients	Household-level variables: Average daily quantitative household consumption of 12 food groups and 27 nutrients from all sources combined (purchased, produced at home, and received as gifts).		**√**	**√**	**√**	**√**	**√**	**√**	
Individuals’ self-evaluation of nutrition knowledge and its application to their lives	Individual-level variables: “Qualitatively evaluate your bodyweight”; “Do you know of and understand the Mongolian national dietary guidelines?”; “Do you understand the importance of dietary diversity?”, “Do you understand the importance of eating regularly?”; “Do you try to cook with and eat less sugar and sugary foods, less fat and fatty foods, more fresh foods, more fruits, and more vegetables?”, “Do you understand what a healthy and balance diet is?”; “How would you evaluate the quality your diet?”; “Do you understand that nutrition is important for health maintenance, or for your child’s health?”, “How important is your nutrition knowledge to your health?”; “How do you evaluate your nutrition knowledge?”; “Do you pay attention to each of the following: nutrition facts, ingredient labels, health claims, expiration dates”; “Have you attended any nutrition training?”; “Do you take any nutritional supplements?”.			**√**	**√**	**√**	**√**	**√**	
Cursory qualitative 24-h recall and assessment of eating behaviors	Individual-level variables: Binary (yes or no) consumption of 12 food groups yesterday; “Did you ever out in the past year?”; “Did you skip any meals in the past 2 days?”; “Did you miss any meals with your family yesterday?”; “Did you eat more, less, or the same amount today as yesterday?”; “Did you eat any foods outside home yesterday?”; “Did you miss any meals yesterday (breakfast, lunch dinner)?”; “Did you eat any snacks yesterday?”.				**√**				
Cursory semiquantitative 24-h recall and assessment of eating behaviors	Individual-level variables: Number of foods eaten yesterday from each of 12 food groups; frequency of snack consumption and eating out in the past year; number of meals (breakfast, lunch, dinner) skipped in last 2 days; “Did you eat more, less, or the same today as yesterday?”; sum of meals (breakfast, lunch, dinner) eaten with family yesterday; total number of food items eaten in each of the following places yesterday: home, outside, someone else’s house, elsewhere; total number of food items eaten yesterday for each meal (breakfast, lunch, dinner) and as snacks.					**√**			
Detailed semiquantitative 24-h recall	Individual-level variables: Binary (yes or no) consumption of 136 different foods during the past 24 h.						**√**	**√**	
Measured anthropometry	Individual-level variables: Measured height and weight; body-mass index; measured waist, hip, mid-arm, and wrist circumference.							**√**	

Models designated 1–5 correspond to those described in detail in Table 3 and Table 4. Brief description of variable categories considered for selection in each model: (1) Household and individual demographic, socioeconomic, and lifestyle characteristics; (2) Model 1 variables + quantitative total household consumption of food groups and nutrients; (3) Model 2 variables + individuals’ self-evaluation of nutrition knowledge and its application to their lives; (4a) Model 3 variables + cursory qualitative 24-h recall and assessment of eating behaviors; (4b) Model 3 variables + cursory semiquantitative 24-h recall and assessment of eating behaviors; (4c) Model 3 variables + detailed semiquantitative 24-h recall; (5) Model 4 variables + measured anthropometry. Number of observations analyzed in each model: 1 and 2 (1282); 3 and 4b (1142); 4a (1140); 4c (1129); 5 (1056). Abbreviation: FCS-24 (nested 24-h recall of the 2013 Food Consumption Survey).

## Supplementary Materials

The following are available online at http://www.mdpi.com/2072-6643/10/6/703/s1, Figure S1: Relationship between per-capita household energy consumption and per-capita dietary intake (kcal/day) among 109 FCS-HH households fully-enumerated by the nested FCS-24 (Aim 1), Figure S2: Mean bias of disaggregated household consumption estimates of individuals’ food group and selected nutrient intake and intake density (per 100 kcal) across 14 age-sex groups (Aims 2 and 3), Figure S3: In-sample fit statistics for increasingly complex predictive models of individuals’ dietary intakes densities of selected nutrients in the FCS-24 (Aim 4), Table S1: Schofield equations for predicting individuals’ basal metabolic rate, Table S2: Ratios of within- to between-person components of variance in dietary nutrient intakes within subgroups of men and women in urban and rural Mongolia, Table S3: Percentage of individuals or households observed to consume any of each food group or nutrient during each survey’s reference period, Table S4: Correlations between ‘ total daily household consumption in the FCS-HH (upper) and between individuals’ daily dietary intakes in the FCS-24 (lower), Table S5: Mean per-capita dietary intakes and intake densities (per 100 kcal), household consumption and consumption densities (per 100 kcal), and correlation between dietary-derived and household-derived per-capita measurements among 109 FCS-HH households fully-enumerated in the nested FCS-24 (Aim 1), Table S6: Goodness of fit statistics for statistical disaggregation models of household food group and nutrient consumption, Table S7: Mean bias of household disaggregation methods in estimating ranks of food group and nutrient intakes and intake densities (per 100 kcal) across 14 age-sex groups (Aims 2 and 3), Table S8: In-sample fit statistics for increasingly complex predictive models of individuals’ dietary intakes and intake densities (per 100 kcal) in the FCS-24 (Aim 4), Table S9: Mean absolute error of alternate prediction methods of individuals’ dietary intake densities of nutrients in the FCS-24: direct prediction of nutrient densities (left) vs. estimation based on separate prediction of nutrient intake and energy intake (right) (Aim 4).

## Appendix A. Details of Multiple Pass 24-h Recall

The 24-h recall consisted of the following passes: (1) an un-ordered “quick list” of the previous day’s foods, along with times and activities during which they were consumed; (2) a probe for potential forgotten foods; (3) detailed descriptions of each food, including ingredients, brands, and preparation methods; (4) detailed assessment of portion sizes; and (5) an ordered review of reported foods and final check for forgotten foods. Portion sizes (pass 4) were assessed using a book of photographs with 3 sections: (1) photos of different vessels and containers (supplemented by physical cups, plates, bowls, and spoons as needed); (2) photos of different Mongolian foods; and (3) photos of vessels filled with different fractions of each food; portion masses could thus be estimated by multiplying the fraction provided in Section 3 by the mass of the food provided in Section 2 that would be equivalent to the full volume of the vessel provided in Section 1. Foods, ingredients, and descriptions included in the photo book and suggested by the various prompts and probes were informed by the experience of the dieticians developing the 24 h. The recall was carried out by 6 teams of 3 individuals each (1 team leader experienced in community health surveys and 2 dietetics students, the former of whom administered the accompanying household consumption questionnaire). After two days of training (during which the dietetics students practiced administering the recall on one another), the teams conducted a pilot study among 100 households.

## Appendix B. Creation of Food Groups, Age Groups, and Other Derived Variables

Due to differences in the descriptiveness and granularity with which household food consumption and individual dietary intake were assessed across the two household surveys and the 24HR, foods reported in the FCS-HH, nested FCS-24, and HSES-HH were condensed into 12 broad food groups to allow more interpretable comparisons across surveys. One of these food groups, “Miscellaneous foods”, was fairly small and its composition differed markedly across datasets, and was therefore omitted from further analyses. To conserve statistical power in disaggregation analysis of the smaller FCS-HH, individuals in all three surveys were condensed into 10 age groups: 0–4, 5–9, 10–14, 15–19, 20–29, 30–39, 40–49, 50–59, 60–69, and 70+ years. For use in regression models, variables were created in the FCS-HH and HSES-HH to quantify each individual’s total daily predicted caloric requirement, and each household’s total daily predicted caloric requirement separately attributable to the person-time of permanent and impermanent members present in the household (impermanent members were defined as registered household members—distinguished from guests and visitors—who reported at least one day away from the household during the reference period). These caloric variables were created using information on household members’ time spent away from home and equations for predicting individuals’ basal metabolic rate and caloric requirement which incorporated age, sex, height, weight, and physical activity categories (Table S1) [49,63]. As information on physical activity was not available in the HSES-HH, trimmed mean values of physical activity levels were estimated for individuals of different strata defined by age, sex, and urban vs. rural locality in the FCS-HH and assigned to individuals in corresponding strata in the HSES-HH. A categorical variable was also created for the FCS-HH and HSES-HH to describe households’ family composition, distinguishing households with one adult male only, one adult female only, multiple adults, multiple adults and one or more children, children only, and no permanent members.

## Appendix C. Derivation and Application of Survey Weights

The FCS-HH, nested FCS-24, and HSES-HH study populations differ between one another (and between the Mongolian national population) in their distributions of variables expected to influence or otherwise associate with household food consumption and individual dietary intake. Survey weights were applied to (1) allow better comparability between disaggregated household consumption estimates obtained from the FCS-HH and HSES-HH household surveys and individual dietary intake in the FCS-24 (Aims 2 and 3); (2) derive more nationally-representative ingredient cooking yield factors from the FCS-24 for calculation of household nutrient consumption in the FCS-HH and HSES-HH (Appendix E); and (3) derive more nationally-representative components of variance in dietary nutrient intake from repeated days of diet records with which to adjust the FCS-24 measurements (Appendix F). For these purposes, weights were calculated for the FCS-HH, nested FCS-24, pooled independent samples of the 2012 and 2014 survey waves of the HSES-HH, and the nationwide diet records using reference data on the distribution of households across 42 strata defined by urban vs. rural locality and national province in the national census of Mongolia [60]. Weights were generated using the national census rather than the FCS-HH as the reference population in order to provide more nationally-representative estimates, and replaced those previously generated for households and individuals in the FCS-HH and HSES-HH in order to maintain comparability both between analyses of households and individuals and between analyses of the FCS-HH, nested FCS-24, HSES-HH, and diet records.

## Appendix D. Adjustment of Household Food Consumption Measurements

For each food within each food group, reported total household consumption (in the FCS-HH and HSES-HH) and individuals’ dietary intake (in the FCS-24) in grams, kilograms, pieces, or liters over the reference period was converted to grams per day, using food densities and mass equivalents as necessary [64]. Daily masses consumed were adjusted as needed for refuse factors [65] and an estimated wastage and spoilage factor of 10% for all foods as recommended by the Ministry of Agriculture, Fisheries, and Food [66]. Because the household consumption data analyzed in this study (and those of most household surveys) only account for consumption of foods originating from home supplies, FCS-HH and HSES-HH household consumption measurements of each food group was adjusted in an attempt to account for foods originating from outside home, using factors estimated for each food group within each urban and rural area of each national province. These stratum-specific factors were calculated using information from the FCS-24, namely the source of each food consumed (home vs. away from home), by computing a stratum-specific trimmed mean of the fraction of each food group sourced from outside of home and adding this fraction to each household’s consumption of the corresponding food group in the corresponding stratum (this may be considered a food group-specific version of one of two approaches suggested by Chesher in statistical disaggregation of household energy consumption [18]).

## Appendix E. Calculation of Dietary Nutrient Intake and Total Household Nutrient Consumption

Daily consumption of 27 nutrients was calculated for individuals participating in the FCS-24 using a purpose-built food composition table previously compiled for Mongolian children, which included analyses of a limited number of local food samples [67]. This table was updated with local data on recipes and dish yields [22,68], incorporating international food composition data primarily from the United States (U.S.) and Germany [65,69] after adjustment for differences in moisture and fat content [68] and application of nutrient retention factors [70] as appropriate. These data were also updated with refuse factors for analysis of the FCS-24 [65]. Calculation of total daily nutrient consumption by households in the FCS-HH and HSES-HH began with each household’s total daily food consumption adjusted for refuse, waste and spoilage, and eating out, and was further adjusted for changes in ingredient mass which occur during cooking of household foods (this was necessary because consumption of household ingredients was reported in terms of raw masses which were not immediately comparable with those reported in the 24HR). To do this, each ingredient consumed in the FCS-24 was first coded with a yield factor specific to each combination of ingredient and the food item that it was found in; the majority of these factors were empirical dish yields derived using locally-collected recipes [22], supplemented as appropriate with ingredient cooking yields from the U.S. and Germany [71,72,73]. A survey-weighted average yield for each ingredient across all instances of its consumption in the FCS-24 was then calculated and used to adjust consumption of corresponding ingredients in both household surveys. Household nutrient consumption was then computed using the same food composition data used for analysis of the FCS-24. Calculated dietary energy intake and household energy consumption, dietary intake and household consumption of food groups and nutrients were also expressed in “energy-adjusted” terms (per 100 kcal of intake or consumption, respectively) to produce “intake densities” and “consumption densities”, respectively [28].

## Appendix F. Adjustment of Dietary Nutrient Intakes for Within-Person Variance

Regardless of sample size, data from a single 24HR per person do not provide accurate estimates of between-person variation in long-term diet [74]. The true distribution of dietary nutrient intake in each of four strata defined by urban vs. rural area and sex was estimated by adjusting each individual’s observed intake according to an empirical Bayesian method, by which an individual’s long-term dietary intake is expressed as a weighted average of their observed estimate and the stratum mean, in which the weights are strata-specific ratios of within- to between- person components of variance [75] (this method is comparable to the National Research Council method [74]). to For this purpose, stratum- and nutrient-specific variance components were first estimated by analyzing nutrient intake data on 6 days of weighed diet records (3 in summer and 3 in winter) collected from 80 healthy Mongolian adults in each of four strata defined by urban vs. rural locality and sex [22], using a survey-weighted fixed effects model in which the logarithm of daily nutrient intake was regressed upon the variables participant ID, season, and day type (weekday vs. weekend day) (SAS v9.4 ‘glm’ procedure). Five of 1839 available person-days of observation for which the ratio of daily total energy intake (TEI) to daily total energy expenditure (TEE) lay beyond 3 standard deviations of the grand (all-strata) median were excluded prior to partitioning variance following a comparable approach in the literature [23], followed by exclusion of 4 person-days of similarly extreme dietary intake (> or <3SD) of each nutrient being considered. Estimated ratios of within- to between-person variance for each nutrient and stratum are given in Table S2.

Differences in the ways that foods and ingredients were expressed in the FCS-24 and diet records prohibited this variance adjustment for food groups. Statistics which rely on accurate estimates of between-person variation in individuals’ dietary intakes (namely, coverage probability of disaggregated household consumption estimates (Aims 2 and 3) and mean absolute bias and percentage of deviance explained in prediction of individual dietary intakes and intake densities (Aim 4)) are therefore estimated exclusively for nutrients. Furthermore, given the lack of repeated measures data for household nutrient consumption, observed household consumption of both food groups and nutrients is similarly unadjusted for within-household variance. However, resulting imprecision is expectedly mitigated by the fact that these household consumption estimates are in fact averages of 7, 10, or 30 day-long reference periods (the relative stability of household estimates is partly supported by the higher observed correlations between household consumption of different foods, particularly nutrients (Table S4)).

## Appendix G. Equations for Describing the Statistical and AME Disaggregation Methods

### Appendix G.1. Statistical Method

Let:

- HC_i_ = total household consumption of a food group or nutrient.
- X_ij_ = the number of persons in the j-th age-sex group within the i-th household (j = 1, …, 20).
- Z_i_ = a vector of covariates (education, family composition, locality, outside food consumption, caloric contribution of impermanent members).
- *l* = 1012 or 9424 households in the FCS-HH and HSES-HH, respectively.

Regression was run of the following form using a Tweedie response distribution, identity link function, and one of 21 possible values of the Tweedie index parameter *p* (ranging from 1 to 3 in increments of 0.1) which produced the smallest ratio of residual to null deviance:

(A1) $$HC_{i}=\alpha +\overset{20}{\underset{j=1}{\sum }}\beta_{j}X_{\mathrm{ij}}+\overset{5}{\underset{k=1}{\sum }}\gamma_{k}Z_{\mathrm{ik}},\;i=1,\ldots ,l$$

In the unadjusted statistical method (“SD1”), the m-th individual’s dietary intake in the i-th household (y_im,*SD1*_) was set to their age- and sex-specific parameter estimate β_jm_ (the latter of which were first smoothed across age groups within each sex using regression splines):

y_im,*SD1*_ = β_jm_ (A2)

In the alternate “AME-like” statistical method (“SD2”), individuals’ dietary intakes y_im,*SD2*_ were instead calculated as follows:

(A3) $$y_{im,\;SD2}=HC_{i}*\;\beta_{jm}/\overset{n_{i}}{\underset{m=1}{\sum }}\beta_{jm},\;i=1,\ldots ,l$$

### Appendix G2. AME Method

Let C_im_ = caloric requirement for the m-th person in the i-th household (Table S1).

Individuals’ dietary intakes y_im,*AME*_ were calculated as follows:

(A4) $$y_{im,\;\;AME}=HC_{i}*\;C_{im}/\overset{n_{i}}{\underset{m=1}{\sum }}C_{im},\;i=1,\ldots ,l$$
