# Supplementary material for: Comparison of Methods for Estimating Dietary Food and Nutrient Intakes and Intake Densities from Household Consumption and Expenditure Data in Mongolia
Source: Nutrients. 2018 May 31;10(6):703. doi: 10.3390/nu10060703 (PMC6024672; doi:10.3390/nu10060703)
Supplement: Supplementary file 1 [file nutrients-10-00703-s001.zip › nutrients-297930-SI/Figure 1, Revised.pdf]

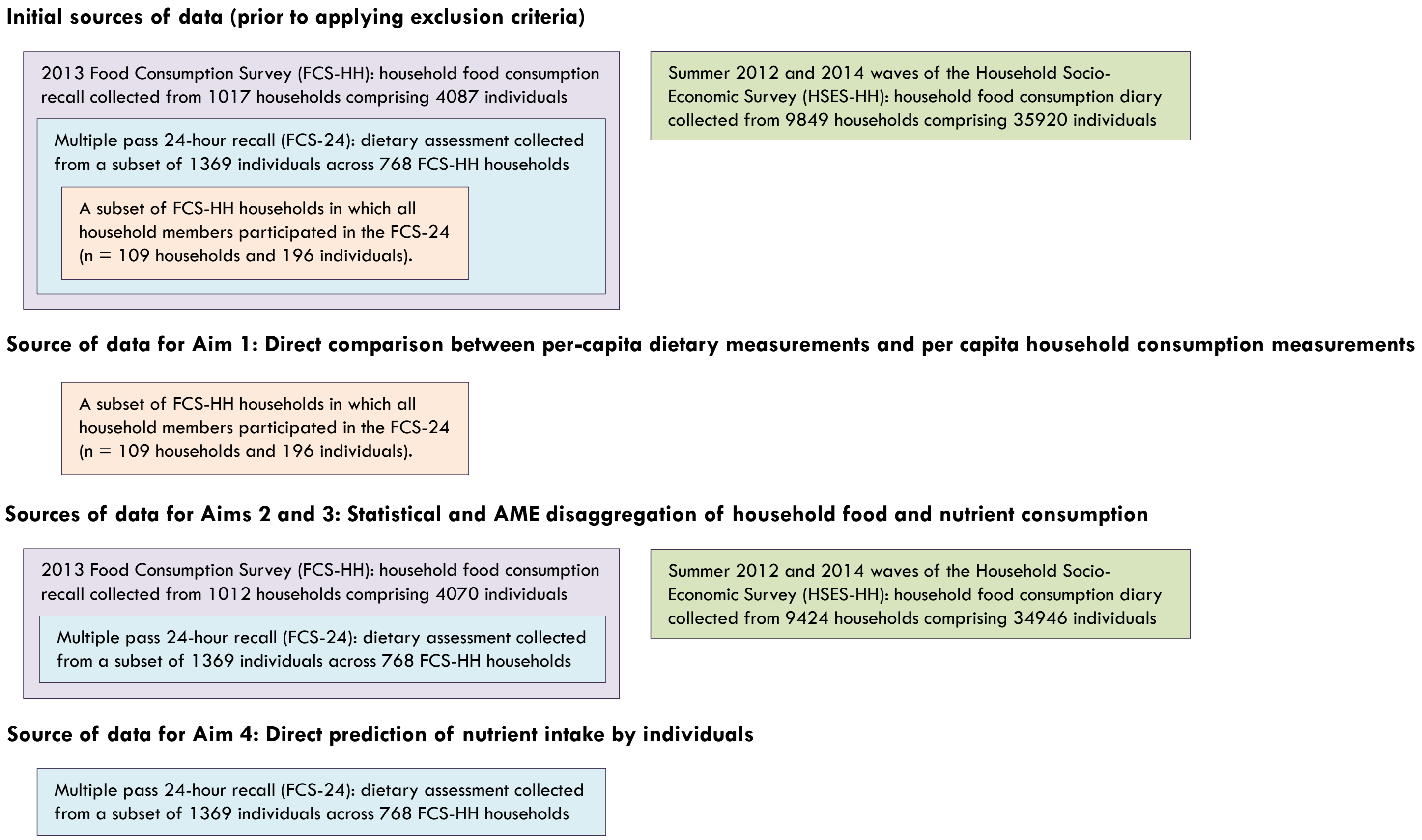

Figure 1. Sources of data. See Methods for description of exclusion criteria and information regarding ancillary diet records and national census data used in preparing and analyzing the FCS-HH, nested FCS-24, and HSES-HH.
