# Supplementary material for: Comparison of Methods for Estimating Dietary Food and Nutrient Intakes and Intake Densities from Household Consumption and Expenditure Data in Mongolia
Source: Nutrients. 2018 May 31;10(6):703. doi: 10.3390/nu10060703 (PMC6024672; doi:10.3390/nu10060703)
Supplement: Supplementary file 1 [file nutrients-10-00703-s001.zip › nutrients-297930-SI/Figure 2.pdf]

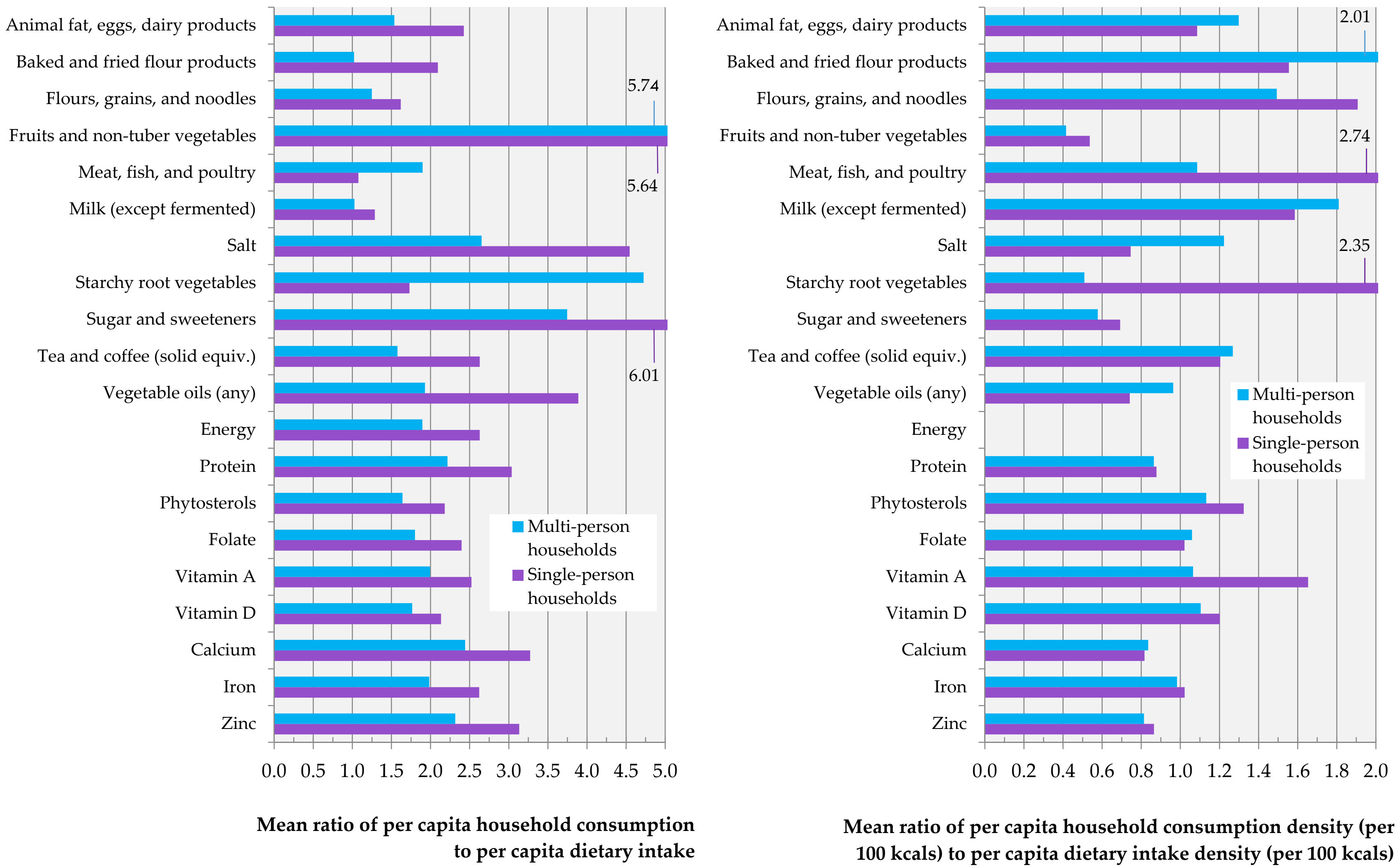

Figure 2. Mean ratio of per-capita household consumption to per-capita dietary intake (left), and per-capita household consumption density (per 100 kcal) to per-capita dietary intake density (per 100 kcal) (right) for food groups and selected nutrients among 109 FCS-HH households fully-enumerated by the nested FCS-24 (Aim 1). Values for 6 data points exceed the graphs' x-axis limits and are indicated using annotations. Abbreviations: FCS-HH (2013 Food Consumption Survey), FCS-24 (nested 24-hour recall).
