# Supplementary material for: Comparison of Methods for Estimating Dietary Food and Nutrient Intakes and Intake Densities from Household Consumption and Expenditure Data in Mongolia
Source: Nutrients. 2018 May 31;10(6):703. doi: 10.3390/nu10060703 (PMC6024672; doi:10.3390/nu10060703)
Supplement: Supplementary file 1 [file nutrients-10-00703-s001.zip › nutrients-297930-SI/Figure 3.pdf]

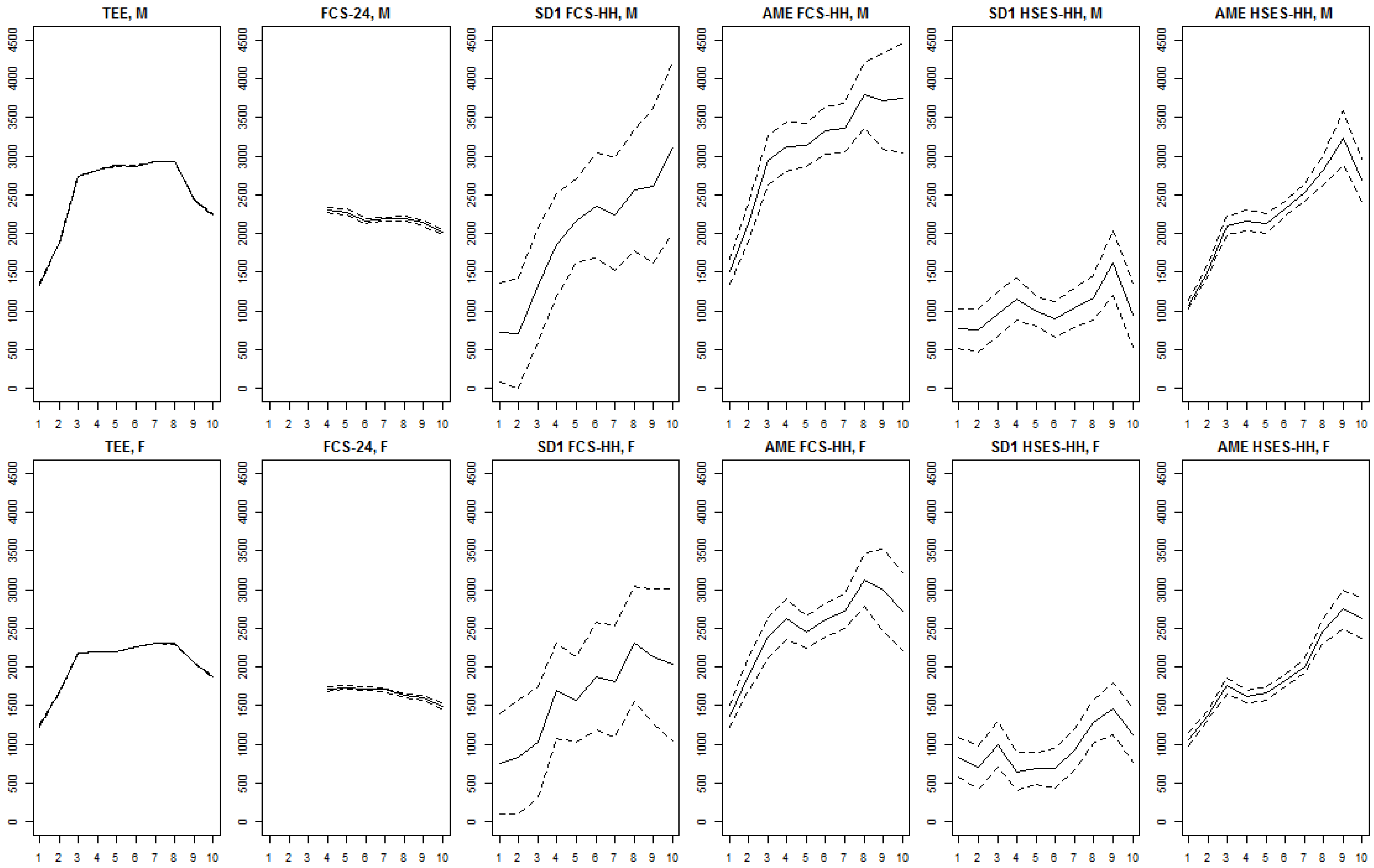

Figure 3. Comparison between mean daily estimated energy expenditure, observed dietary energy intake, and disaggregated household consumption estimates of individuals' energy intake (kcal/day) across 10 age groups of males and females (Aims 2 and 3). **y-axis:** daily energy intake (kcal/day); **x-axis:** age groups 1-10 (1: 0 to 4 years, 2: 5-9, 3: 10-14, 4: 15-19, 5: 20-29, 6: 30-39, 7: 40-49, 8: 50-59, 9: 60-69, 10: 70+); **row 1:** males ("M"); **row 2:** females ("F"); **column 1:** mean predicted total energy expenditure ("TEE"), **column 2:** mean observed dietary energy intake from the FCS 24-hour recall ("FCS-24"); **column 3:** unadjusted statistical disaggregation of FCS-HH ("SD1 FCS-HH"); **column 4:** AME-like statistical disaggregation of FCS-HH ("SD2 FCS-HH"); **column 5:** AME disaggregation of FCS-HH ("AME FCS-HH"); **column 6:** unadjusted statistical disaggregation of HSES-HH ("SD1 HSES-HH"); **column 7:** AME-like statistical disaggregation of HSES-HH ("SD2 HSES-HH"); **column 8:** AME disaggregation of HSES-HH ("AME HSES-HH"). **Solid lines** indicate means of age- and sex-specific measurements (FCS-24) or predictions (TEE and disaggregated household estimates), while **dashed lines** indicate associated 95% confidence limits. Statistics *are* survey-weighted. Abbreviations: FCS-HH (2013 Food Consumption Survey), HSES-HH (2012/2014 Household Socio-Economic Survey), AME (adult male equivalent method).
