# Supplementary material for: Comparison of Methods for Estimating Dietary Food and Nutrient Intakes and Intake Densities from Household Consumption and Expenditure Data in Mongolia
Source: Nutrients. 2018 May 31;10(6):703. doi: 10.3390/nu10060703 (PMC6024672; doi:10.3390/nu10060703)
Supplement: Supplementary file 1 [file nutrients-10-00703-s001.zip › nutrients-297930-SI/Figure 4.pdf]

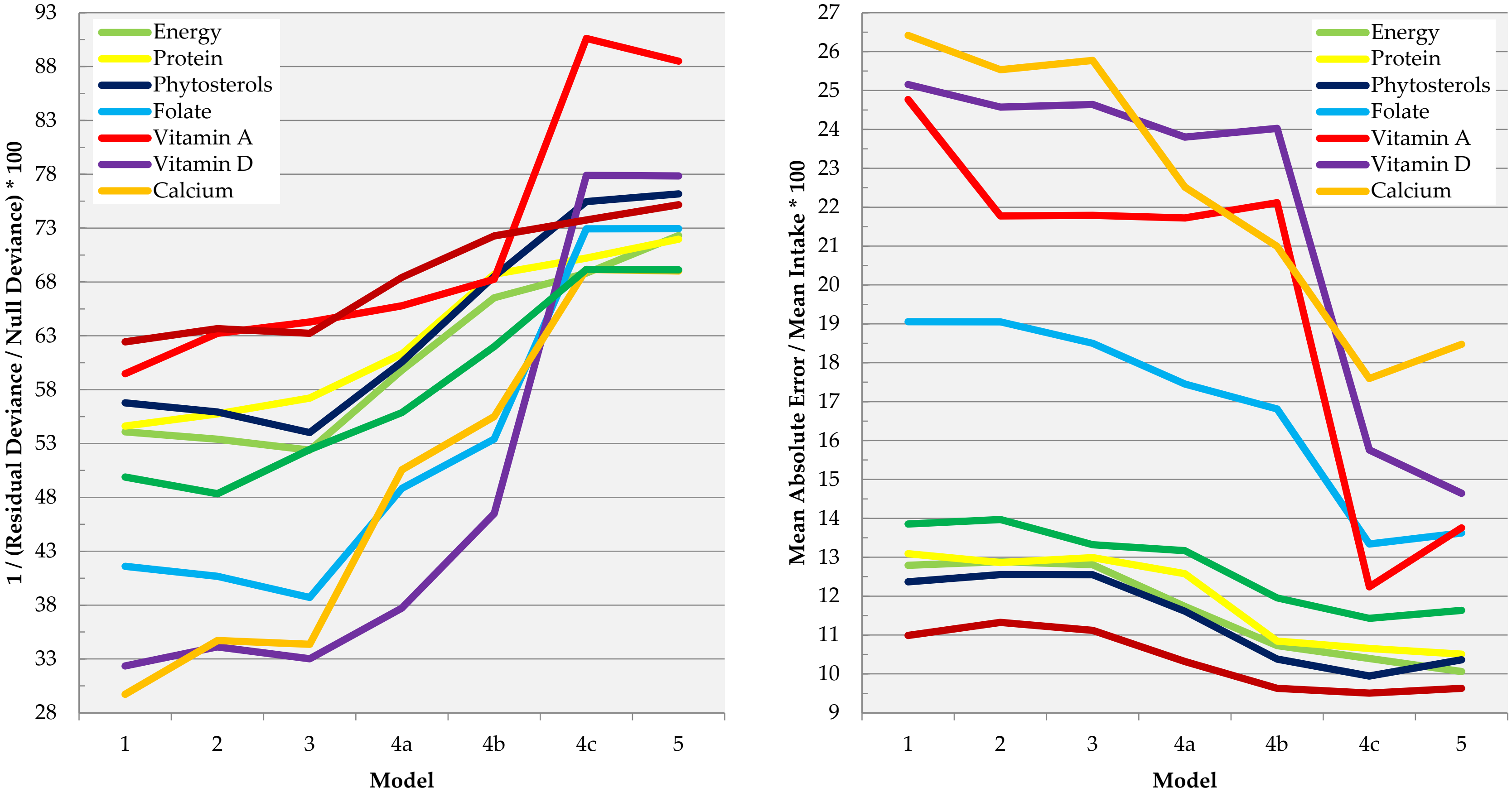

Figure 4. In-sample fit statistics for increasingly complex predictive models of individuals' dietary intakes of selected nutrients in the FCS-24 (Aim 4). See Table 3.4 for detailed descriptions of models 1-5. Brief description of variable categories considered for selection in each model: (1) Household and individual demographic, socioeconomic, and lifestyle characteristics, (2) Model 1 variables + quantitative total household consumption of food groups and nutrients, (3) Model 2 variables + individuals' self-evaluation of nutrition knowledge and its application to their lives, (4a) Model 3 variables + cursory qualitative 24-hour recall and assessment of eating behaviors, (4b) Model 3 variables + cursory semiquantitative 24-hour recall and assessment of eating behaviors, (4c) Model 3 variables + detailed semiquantitative 24-hour recall, (5) Model 4 variables + measured anthropometry. Abbreviation: FCS-24 (nested 24-hour recall of the 2013 Food Consumption Survey).5
