# Supplementary material for: Comparison of Methods for Estimating Dietary Food and Nutrient Intakes and Intake Densities from Household Consumption and Expenditure Data in Mongolia
Source: Nutrients. 2018 May 31;10(6):703. doi: 10.3390/nu10060703 (PMC6024672; doi:10.3390/nu10060703)
Supplement: Supplementary file 1 [file nutrients-10-00703-s001.zip › nutrients-297930-SI/nutrients-297930-sup.pdf]

Supplementary Materials: Comparison of methods for estimating dietary food and nutrient intakes and intake densities from household consumption and expenditure data in Mongolia

Sabri Bromage, Bernard Rosner, Janet W. Rich-Edwards, Davaasambuu Ganmaa, Soninkhishig Tsolmon, Zuunnast Tserendejid, Tseye-Oidov Odbayar, Margaret Traeger, and Wafaie W. Fawzi

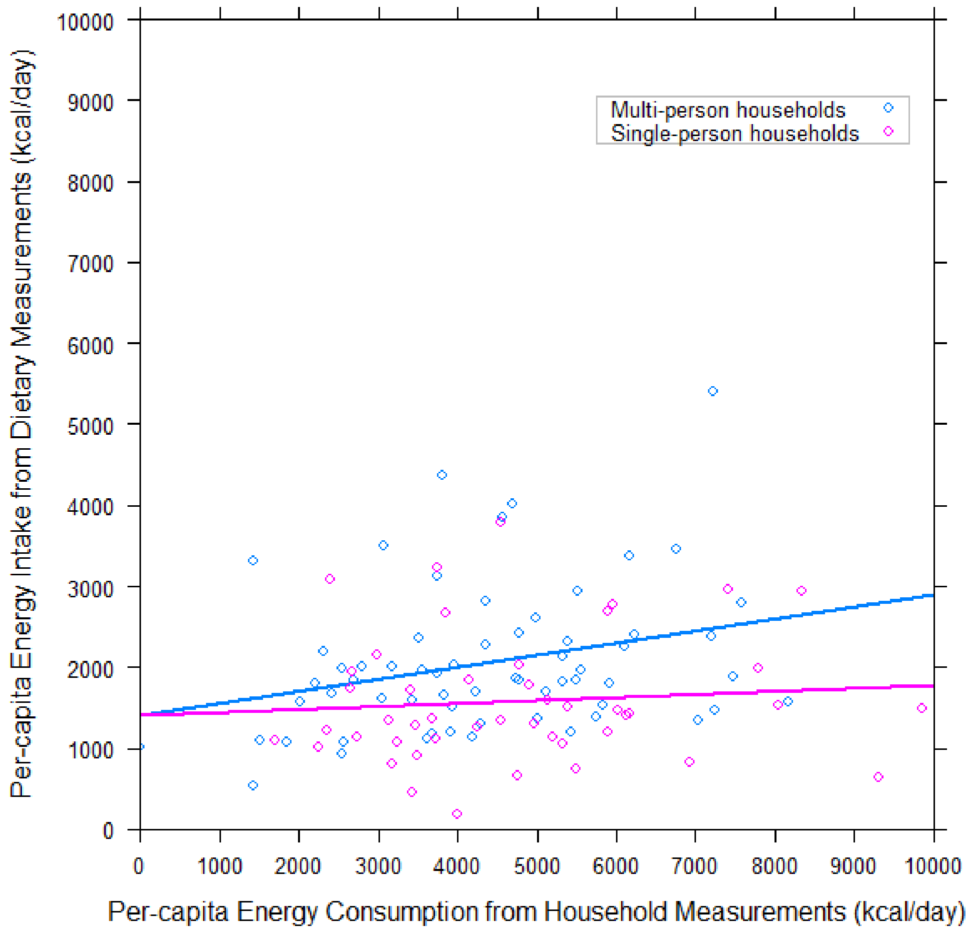

Figure S1. Relationship between per-capita household energy consumption and per-capita dietary intake (kcal/day) among 109 FCS-HH households fully-enumerated by the nested FCS-24 (Aim 1). Pearson correlation coefficients for multi- and single-person households: 0.29 and 0.09, respectively (Spearman rank correlation coefficients: 0.29 and 0.14, respectively).

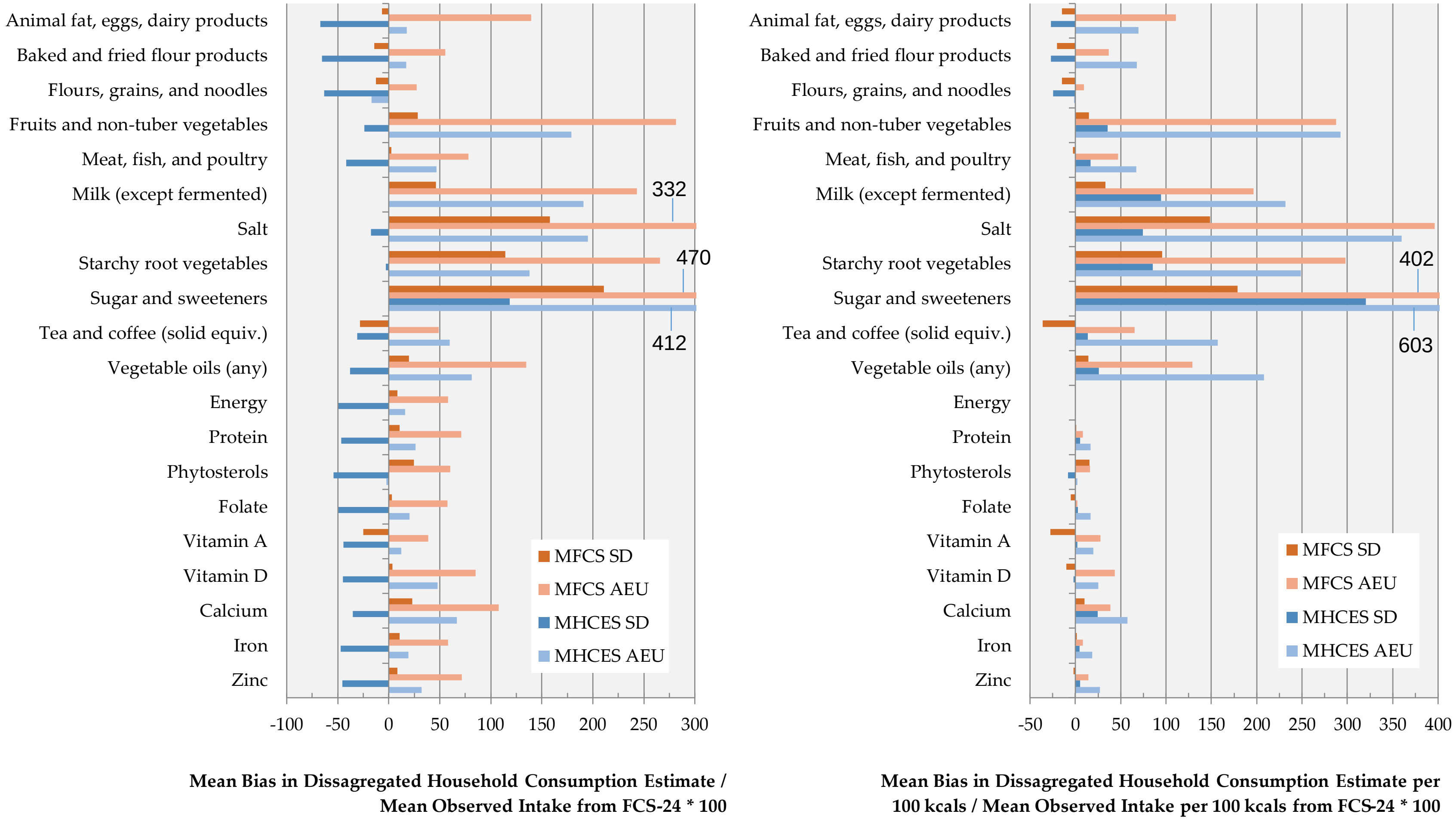

Figure S2. Mean bias of disaggregated household consumption estimates of individuals' food group and selected nutrient intake and intake density (per 100 kcal) across 14 age-sex groups (Aims 2 and 3). Values for 5 data points exceed the graphs' x-axis limits and are indicated using annotations. Abbreviations: FCS-HH (2013 Food Consumption Survey), FCS-24 (nested 24-hour recall), HSES-HH (2012/2014 Household Socio-Economic Survey), SD1 (unadjusted statistical disaggregation method), AME (adult male equivalent method). Statistics are survey weighted.

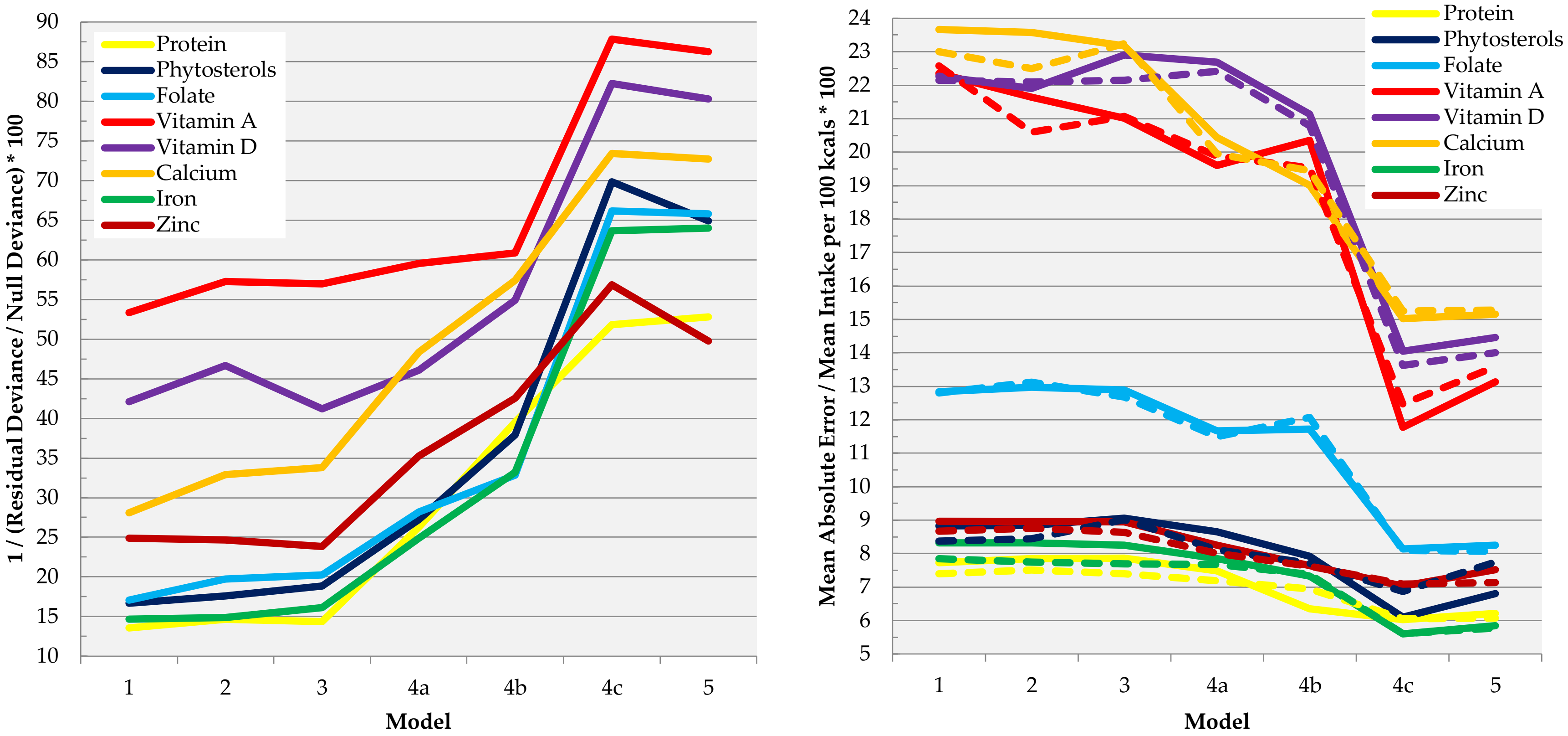

Figure S3. In-sample fit statistics for increasingly complex predictive models of individuals' dietary intakes densities of selected nutrients in the FCS-24 (Aim 4). Panel A: percentage of deviance explained; Panel B: mean absolute bias (proportional to mean observed dietary intake). In the right panel, dashed lines indicate mean absolute error estimated by separately predicting nutrient intake and energy intake, dividing predicted nutrient intake by predicted energy intake, and comparing the results to observed dietary intake measurements in the FCS-24, while solid lines indicate mean absolute error of predicting nutrient intake densities directly. See Table 4 for detailed descriptions of models 1-5. Brief description of variable categories considered for selection in each model: (1) Household and individual demographic, socioeconomic, and lifestyle characteristics, (2) Model 1 variables + quantitative total household consumption of food groups and nutrients, (3) Model 2 variables + individuals' self-evaluation of nutrition knowledge and its application to their lives, (4a) Model 3 variables + cursory qualitative 24-hour recall and assessment of eating behaviors, (4b) Model 3 variables + cursory semiquantitative 24-hour recall and assessment of eating behaviors, (4c) Model 3 variables + detailed semiquantitative 24-hour recall, (5) Model 4 variables + measured anthropometry. Abbreviation: FCS-24 (nested 24-hour recall of the 2013 Food Consumption Survey)

Table S1. Schofield equations for predicting individuals' basal metabolic rate

Sex	Age	Equation
Male	0-3 yrs	0.0007 * W + 6.349 * H - 2.584
	3-10 yrs	0.082 * W + 0.545 * H + 1.736
	10-18 yrs	0.068 * W + 0.574 * H + 2.157
	18-30 yrs	0.063 * W - 0.042 * H + 2.953
	30-60 yrs	0.048 * W - 0.011 * H + 3.670
	60+ yrs	0.038 * W + 4.068 * H - 3.491
Female	0-3 yrs	0.068 * W + 4.281 * H - 1.730
	3-10 yrs	0.071 * W + 0.677 * H + 1.553
	10-18 yrs	0.035 * W + 1.948 * H + 0.837
	18-30 yrs	0.057 * W + 1.184 * H + 0.411
	30-60 yrs	0.034 * W + 0.006 * H + 3.50
	60+ yrs	0.033 * W + 1.917 * H - 0.074

Table is reproduced from Schofield 1985. Total energy expenditure is estimated by multiplying basal metabolic rate by the mean of a range of coefficients suggested for different categories of physical activity level: sedentary or light activity (1.40-1.69), active or moderately active (1.70-1.99), vigorous or vigorously active (2.00-2.40) (FAO 2005).

Table S2. Ratios of within- to between-person components of variance in dietary nutrient intakes within subgroups of men and women in urban and rural Mongolia

	**Area:**	**Rural**		**Urban**	
	**Sex:**	**Female**	**Male**	**Female**	**Male**
**Macronutrients**	Energy	2.1	2.5	1.2	2.9
	Carbohydrates	2.7	1.7	1.0	3.1
	Protein	1.8	3.2	2.1	3.5
	Total fat	1.9	2.1	1.5	2.9
	Alcohol	3.3	1.5	7.9	3.5
	Water	1.2	0.9	1.0	1.2
	Fiber	2.9	2.5	1.5	3.6
	Phytosterols	3.8	3.3	1.7	4.4
**Vitamins**	Thiamin	2.3	1.6	1.5	2.3
	Riboflavin	1.6	1.8	1.9	3.2
	Niacin	3.6	4.0	2.7	2.5
	Pantothenic acid	1.5	2.4	1.8	4.4
	Vitamin B6	1.6	1.3	2.7	2.9
	Folate	1.7	1.2	1.6	2.3
	Vitamin B12	4.9	3.5	7.0	4.1
	Vitamin C	4.1	20.9	8.5	5.3
	Vitamin A	2.2	2.8	4.2	3.8
	Vitamin D	1.8	1.7	2.9	6.5
	Vitamin E	2.9	1.8	1.8	9.0
**Minerals**	Calcium	1.0	0.9	1.6	1.8
	Copper	2.1	2.8	2.6	3.7
	Iron	2.3	1.7	1.8	3.0
	Magnesium	1.5	2.1	1.7	3.2
	Manganese	1.6	2.3	1.3	3.7
	Phosphorus	2.0	1.9	1.5	2.9
	Potassium	1.6	2.5	2.3	3.9
	Zinc	2.5	6.0	3.1	3.3

Components of variance are derived from analysis of 3-day summer and winter diet records collected from 320 Mongolian adults living in urban and rural Mongolia, and are survey-weighted.

Table S3. Percentage of individuals or households observed to consume any of each food group or nutrient during each survey's reference period

	Survey:	FCS-24	FCS-HH	HSES-HH
	n:	1368 individuals	1017 households	9849 households
	Length of Reference Period:	1 day	7 to 30 days	7 to 10 days
Food Groups	Animal fat, eggs, and dairy products	53.5	95.6	87.8
	Baked and fried flour products	83.3	94.6	85.1
	Flours, grains, and noodles	94.6	100.0	99.8
	Fruits and non-tuber vegetables	62.6	95.6	90.0
	Meat, fish, and poultry	97.1	99.5	99.9
	Milk (except fermented)	71.6	95.4	94.1
	Salt	82.3	98.0	98.6
	Starchy root vegetables	53.8	93.2	82.5
	Sugar and sweeteners	20.1	98.6	98.4
	Tea or coffee (solid equivalent)	49.3	92.5	96.7
	Vegetable oils (any)	41.4	95.6	82.5
Macronutrients	Energy	100.0	100.0	100.0
	Carbohydrates	100.0	100.0	100.0
	Protein	100.0	100.0	100.0
	Total fat	100.0	100.0	100.0
	Alcohol	3.5	20.0	11.3
	Water	100.0	100.0	100.0
	Fiber	99.9	100.0	100.0
	Phytosterols	99.8	100.0	100.0
Vitamins	Thiamin	100.0	100.0	100.0
	Riboflavin	100.0	100.0	100.0
	Niacin	100.0	100.0	100.0
	Pantothenic acid	100.0	100.0	100.0
	Vitamin B6	100.0	100.0	100.0
	Folate	100.0	100.0	100.0
	Vitamin B12	99.9	99.9	100.0
	Vitamin C	96.8	99.9	99.8
	Vitamin A	96.9	99.7	99.4
	Vitamin D	93.8	99.3	99.1
	Vitamin E	100.0	100.0	100.0
Minerals	Calcium	100.0	100.0	100.0
	Copper	100.0	100.0	100.0
	Iron	100.0	100.0	100.0
	Magnesium	100.0	100.0	100.0
	Manganese	100.0	100.0	100.0
	Phosphorus	100.0	100.0	100.0
	Potassium	100.0	100.0	100.0
	Zinc	100.0	100.0	100.0

Statistics are derived after restricting HSES-HH data to those collected in May, June, July, or August, and prior to excluding households with no permanent members or individuals with ratios of total energy intake to expenditure lying 3 standard deviations beyond the median. Shading indicates values less than 100%. Abbreviations: FCS-HH (2013 Food Consumption Survey); FCS-24 (nested 24-hour recall), HSES-HH (2012/2014 Household Socio-Economic Survey.

Table S4. Correlations between ' total daily household consumption in the FCS-HH (upper) and between individuals' daily dietary intakes in the FCS-24 (lower)

Animal fat, eggs, dairy products	Baked and fried flour products	Flours, grains, and noodles	Fruits and non-tuber vegetables	Meat, fish, and poultry	Milk (except fermented)	Salt	Starchy root vegetables	Sugar and sweeteners	Tea and coffee (solid equiv.)	Vegetable oils (any)	Energy	Protein	Folate	Phytosterols	Vitamin A	Vitamin D	Calcium	Iron	Zinc	
1.00	0.21	0.31	0.01	0.30	0.51	0.20	-0.00	0.35	0.12	0.10	0.58	0.60	0.46	0.26	0.51	0.51	0.79	0.35	0.56	Animal fat, eggs, dairy products
	1.00	0.28	0.25	0.31	0.17	0.15	0.15	0.30	0.20	0.14	0.64	0.49	0.80	0.50	0.31	0.35	0.37	0.78	0.44	Baked and fried flour products
		1.00	0.09	0.31	0.22	0.30	0.19	0.44	0.24	0.27	0.73	0.61	0.60	0.80	0.34	0.26	0.33	0.61	0.53	Flours, grains, and noodles
			1.00	0.32	0.01	-0.05	0.26	0.01	0.07	0.23	0.25	0.26	0.31	0.29	0.35	0.34	0.11	0.34	0.23	Fruits and non-tuber vegetables
				1.00	0.23	0.16	0.17	0.30	0.15	0.20	0.65	0.81	0.54	0.41	0.51	0.50	0.37	0.72	0.89	Meat, fish, and poultry
					1.00	0.15	0.02	0.29	0.17	0.12	0.51	0.54	0.34	0.20	0.43	0.58	0.89	0.31	0.49	Milk (except fermented)
						1.00	0.18	0.32	0.20	0.25	0.34	0.27	0.28	0.26	0.22	0.19	0.21	0.27	0.25	Salt
							1.00	0.09	0.05	0.33	0.25	0.20	0.22	0.34	0.21	0.12	0.08	0.27	0.17	Starchy root vegetables
								1.00	0.42	0.17	0.56	0.48	0.49	0.40	0.34	0.29	0.38	0.45	0.45	Sugar and sweeteners
									1.00	0.07	0.31	0.26	0.36	0.26	0.22	0.17	0.18	0.27	0.24	Tea and coffee (solid equiv.)
										1.00	0.33	0.24	0.23	0.28	0.22	0.21	0.18	0.29	0.22	Vegetable oils (any)
											1.00	0.93	0.89	0.75	0.63	0.65	0.69	0.90	0.88	Energy
												1.00	0.78	0.64	0.65	0.68	0.72	0.85	0.96	Protein
													1.00	0.71	0.52	0.51	0.54	0.89	0.73	Folate
														1.00	0.41	0.39	0.34	0.72	0.57	Phytosterols
															1.00	0.58	0.56	0.54	0.59	Vitamin A
																1.00	0.66	0.52	0.63	Vitamin D
																	1.00	0.52	0.65	Calcium
																		1.00	0.82	Iron
																			1.00	Zinc
1.00	0.05	-0.01	0.01	-0.01	0.08	0.00	-0.03	0.17	-0.05	0.01	0.17	0.17	0.18	0.02	0.01	0.06	0.60	0.03	0.10	Animal fat, eggs, dairy products
	1.00	-0.09	-0.05	-0.11	0.05	0.03	-0.04	0.08	-0.02	-0.08	0.34	0.08	0.59	0.16	-0.03	0.12	0.19	0.35	0.01	Baked and fried flour products
		1.00	0.03	0.36	-0.01	0.08	0.26	-0.05	0.05	0.42	0.56	0.49	0.29	0.64	0.11	0.02	0.05	0.41	0.46	Flours, grains, and noodles
			1.00	0.07	-0.04	0.08	0.08	-0.01	0.08	0.09	0.00	-0.01	-0.04	-0.05	0.04	0.05	-0.00	-0.01	0.01	Fruits and non-tuber vegetables
				1.00	-0.02	0.04	0.24	-0.06	0.05	0.15	0.46	0.68	0.26	0.27	0.38	0.19	0.03	0.65	0.72	Meat, fish, and poultry
					1.00	0.08	-0.07	0.08	-0.01	-0.04	0.14	0.12	0.11	0.06	-0.02	0.12	0.53	0.04	0.06	Milk (except fermented)
						1.00	0.02	-0.02	0.03	0.10	0.08	0.05	0.08	0.05	0.02	-0.00	0.05	0.07	0.05	Salt
							1.00	-0.08	0.08	0.21	0.14	0.14	0.03	0.14	0.00	0.04	-0.05	0.12	0.17	Starchy root vegetables
								1.00	-0.01	-0.05	0.06	0.01	0.05	0.00	-0.01	0.01	0.15	0.02	0.01	Sugar and sweeteners
									1.00	0.02	0.00	-0.01	-0.01	-0.00	0.04	0.09	-0.05	0.01	0.01	Tea and coffee (solid equiv.)
										1.00	0.27	0.19	0.07	0.19	0.01	0.01	-0.01	0.16	0.16	Vegetable oils (any)
											1.00	0.87	0.76	0.76	0.18	0.11	0.36	0.84	0.79	Energy
												1.00	0.60	0.65	0.32	0.16	0.35	0.85	0.93	Protein
													1.00	0.57	0.23	0.03	0.32	0.76	0.54	Folate
														1.00	0.11	0.02	0.17	0.62	0.62	Phytosterols
															1.00	0.19	-0.01	0.42	0.43	Vitamin A
																1.00	0.12	0.17	0.13	Vitamin D

1.00	0.22	0.19	Calcium
	1.00	0.83	Iron
		1.00	Zinc

Green-Yellow-Red shading indicates the magnitude of absolute correlation (Green: minimum observed absolute correlation; Yellow: median; Green: maximum). Abbreviations: FCS-HH (2013 Food Consumption Survey); FCS-24 (nested 24-hour recall).

Table S5. Mean per-capita dietary intakes and intake densities (per 100 kcal), household consumption and consumption densities (per 100 kcal), and correlation between dietary-derived and household-derived per-capita measurements among 109 FCS-HH households fully-enumerated in the nested FCS-24 (Aim 1)

	Statistic:	Mean Per-capita Intake or Consumption								Mean Per-capita Intake or Consumption Density (per 100 kcal)							
	Household Type:	Multi-person (n=63)				Single-person (n=46)				Multi-person (n=63)				Single-person (n=46)			
	Derivation of Statistic:	Diet	HH	r_p	r_s	Diet	HH	r_p	r_s	Diet	HH	r_p	r_s	Diet	HH	r_p	r_s
Food Groups	Animal fat, eggs, dairy products (g)	111.9	343.4	0.17	0.35	61.1	231.8	0.37	0.41	5.30	7.35	0.14	0.37	4.05	5.31	0.49	0.32
	Baked and fried flour products (g)	112.5	206.4	0.03	0.07	87.2	219.9	0.25	0.13	5.86	4.50	-0.10	-0.02	5.98	4.66	0.25	0.15
	Flours, grains, and noodles (g)	243.4	409.4	0.02	-0.02	221.7	433.9	0.19	0.19	12.44	9.63	0.12	0.07	14.18	9.38	0.06	0.16
	Fruits and non-tuber vegetables (g)	27.8	124.7	0.50	0.59	20.6	162.4	0.16	0.16	1.39	3.09	0.35	0.61	1.28	3.39	0.15	0.10
	Meat, fish, and poultry (g)	103.3	311.5	0.05	0.19	106.3	433.9	0.25	0.09	5.24	7.11	0.14	0.13	6.83	8.76	0.04	-0.08
	Milk (except fermented) (g)	100.7	447.9	0.16	0.35	78.1	549.5	0.03	0.13	5.23	9.89	0.16	0.26	6.09	11.84	0.24	0.30
	Salt (g)	3.0	10.3	-0.03	0.15	1.6	11.1	0.01	-0.11	0.18	0.26	-0.09	0.24	0.12	0.28	0.38	0.22
	Starchy root vegetables (g)	20.3	110.3	0.12	0.15	25.9	172.2	0.14	0.26	1.04	2.62	-0.02	0.05	1.89	3.68	0.28	0.23
	Sugar and sweeteners (g)	5.4	29.9	0.17	0.20	5.1	23.3	0.28	0.49	0.30	0.67	0.17	0.08	0.43	0.55	0.13	0.40
	Tea and coffee (solid equiv.) (g)	2.6	7.3	0.08	0.00	2.2	8.1	0.10	-0.01	0.16	0.18	0.12	0.07	0.17	0.20	0.29	-0.02
	Vegetable oils (any) (g)	6.7	18.2	0.13	0.05	6.9	27.4	0.16	0.00	0.33	0.45	0.20	0.16	0.45	0.64	0.14	0.00
Macronutrients	Energy (kcal)	2070	4438	0.29	0.29	1583	4767	0.09	0.14	N/A	N/A	N/A	N/A	N/A	N/A	N/A	N/A
	Carbohydrates (g)	262.49	518.88	0.15	0.21	205.51	548.69	0.09	0.09	12.844	11.883	0.08	0.19	13.113	11.682	-0.11	-0.13
	Protein (g)	74.28	189.21	0.28	0.36	60.22	218.59	0.20	0.14	3.633	4.265	0.33	0.32	3.806	4.488	0.08	-0.04
	Total fat (g)	72.54	174.90	0.28	0.37	55.89	186.46	0.01	0.09	3.527	3.857	0.01	0.06	3.505	3.876	-0.11	-0.04
	Alcohol (g)	8.30	1.06	0.64	0.47	0.85	0.89	0.80	0.47	0.258	0.021	0.44	0.47	0.028	0.030	0.89	0.47
	Water (g)	567.40	1785.34	0.25	0.30	473.91	2114.16	-0.03	-0.02	29.664	40.752	0.10	0.09	32.100	46.582	0.17	0.09
	Fiber (g)	9.3	19.2	0.16	0.24	7.1	21.8	0.18	0.10	0.45	0.45	0.22	0.27	0.44	0.45	0.04	-0.04
	Phytosterols (mg)	434	917	-0.06	-0.04	392	1082	0.11	0.11	21.8	21.4	0.20	0.07	24.7	23.2	-0.10	-0.05
Vitamins	Thiamin (mg)	0.780	1.831	0.16	0.15	0.653	2.288	0.25	0.11	0.0396	0.0418	0.08	0.06	0.0432	0.0482	0.10	0.12
	Riboflavin (mg)	1.143	3.557	0.19	0.25	1.012	4.247	0.23	0.05	0.0586	0.0800	0.09	0.13	0.0686	0.0901	0.07	0.16
	Niacin (mg)	12.498	32.558	0.17	0.25	11.479	42.713	0.28	0.24	0.6351	0.7379	0.27	0.28	0.7372	0.8635	0.06	-0.07
	Pantothenic acid (mg)	3.145	8.502	0.31	0.34	2.683	9.943	0.25	0.03	0.1582	0.1942	0.21	0.14	0.1747	0.2107	0.15	0.25
	Vitamin B6 (mg)	0.579	1.565	0.34	0.36	0.514	1.931	0.17	0.18	0.0300	0.0370	0.37	0.44	0.0325	0.0413	0.13	0.23
	Folate (µg)	141	307	0.20	0.26	100	302	0.04	0.00	7.0	6.8	0.13	0.11	6.3	6.4	0.16	0.21
	Vitamin B12 (µg)	4.48	13.69	0.16	0.25	5.24	13.29	0.37	-0.05	0.234	0.317	0.10	0.11	0.335	0.274	0.11	-0.13
	Vitamin C (mg)	8.0	35.8	0.36	0.29	7.6	43.6	0.18	0.26	0.41	0.83	0.20	0.10	0.48	0.91	0.04	0.09
	Vitamin A (µg)	330	900	0.12	0.14	289	728	0.10	0.16	17.0	21.2	-0.08	-0.06	19.4	16.3	-0.06	0.16
	Vitamin D (IU)	26	61	0.27	0.41	40	73	0.35	0.12	1.4	1.3	0.34	0.41	1.8	1.5	0.24	0.10
	Vitamin E (mg)	5.38	12.92	0.17	0.18	4.74	16.85	0.05	-0.09	0.268	0.301	0.20	0.16	0.286	0.377	-0.01	-0.10
Minerals	Calcium (mg)	460	1449	0.26	0.33	338	1615	0.10	0.13	23.2	32.3	0.15	0.28	24.0	35.2	0.29	0.26
	Copper (mg)	0.809	2.097	0.18	0.18	0.827	2.560	0.41	0.27	0.0414	0.0487	0.06	0.10	0.0538	0.0535	0.10	0.27
	Iron (mg)	9.85	22.80	0.20	0.24	8.56	27.44	0.31	0.17	0.498	0.522	0.22	0.22	0.545	0.564	0.07	0.04
	Magnesium (mg)	176	421	0.25	0.23	144	471	0.06	0.01	8.6	9.8	0.45	0.38	8.9	10.0	-0.02	0.02
	Manganese (mg)	2.257	4.791	0.11	0.15	1.797	5.285	0.15	0.10	0.1150	0.1125	0.39	0.37	0.1158	0.1140	0.00	0.25
	Phosphorus (mg)	971	2512	0.27	0.27	750	2857	0.06	-0.03	48.2	56.0	0.20	0.30	48.7	60.0	0.16	0.19
	Potassium (mg)	1405	3997	0.28	0.32	1214	5072	0.04	0.01	69.3	92.1	0.30	0.33	77.3	107.1	0.15	0.13

Zinc (mg)	10.49	29.84	0.13	0.27	9.77	38.27	0.19	0.16	0.514	0.672	0.19	0.22	0.617	0.777	0.09	-0.05

Mean per-capita dietary-derived estimates and mean per-capita household-derived estimates are given in the "Diet" and "HH" columns, respectively. Green-Yellow-Red shading indicates the magnitude of absolute percent difference between household consumption and dietary intake (Green: minimum observed absolute percent difference; Yellow: median; Green: maximum), and Blue-Yellow-Red shading indicates the magnitude of absolute percent difference between household consumption density and dietary intake density (per 100 kcal) (Blue: minimum observed absolute percent difference; Yellow: median; Red: maximum). Abbreviations: FCS-HH (2013 Food Consumption Survey), FCS-24 (nested 24-hour recall), r_P (Pearson correlation coefficient), r_s (Spearman rank correlation coefficient), IU (international unit; 40 IU = 1 μg).

Table S6. Goodness of fit statistics for statistical disaggregation models of household food group and nutrient consumption

	**Household Survey:**	**FCS-HH**			**HSES-HH**		
	**Statistic:**	**% Deviance Explained**	**MAE**	**(Mean HH Consumption)**	**% Deviance Explained**	**MAE**	**(Mean HH Consumption)**
**Food Groups**	Animal fat, eggs, and dairy products (g)	24.8	552.0	687.2	23.1	259.4	303.5
	Baked and fried flour products (g)	12.8	322.3	578.2	19.3	202.2	401.2
	Flours, grains, and noodles (g)	33.9	352.9	1070.6	51.1	251.1	635.2
	Fruits and non-tuber vegetables (g)	25.2	218.1	375.6	12.0	161.7	235.0
	Meat, fish, and poultry (g)	14.2	309.6	679.8	27.2	231.7	531.4
	Milk (except fermented) (g)	28.2	553.9	843.5	34.3	446.0	671.4
	Salt (g)	16.7	12.7	26.6	12.9	7.6	17.3
	Starchy root vegetables (g)	15.3	212.2	365.6	11.3	129.1	206.4
	Sugar and sweeteners (g)	24.5	35.8	67.7	17.9	32.5	53.8
	Tea or coffee (solid equivalent) (g)	9.5	9.7	14.3	10.2	9.5	14.5
	Vegetable oils (any) (g)	13.5	25.9	52.3	7.6	21.4	32.4
**Macronutrients**	Energy (kcal)	35.0	3269	10765	34.1	2418	7191
	Carbohydrates (g)	39.5	386.22	1343.84	42.2	278.32	858.80
	Protein (g)	27.8	147.23	432.05	41.7	105.00	293.08
	Total fat (g)	24.4	156.74	400.55	19.4	120.67	284.42
	Alcohol (g)	10.5	4.42	2.78	2.0	5.32	2.91
	Water (g)	24.2	1480.79	4013.84	33.9	1159.92	2900.21
	Fiber (g)	33.4	15.8	52.6	32.8	10.9	33.0
	Phytosterols (mg)	28.7	849	2464	37.8	488	1394
**Vitamins**	Thiamin (mg)	26.8	1.619	4.844	23.2	1.195	3.472
	Riboflavin (mg)	24.9	3.059	7.803	33.8	2.132	5.493
	Niacin (mg)	23.8	27.576	79.791	28.1	19.840	56.814
	Pantothenic acid (mg)	26.7	6.743	20.356	35.9	5.004	14.181
	Vitamin B6 (mg)	27.8	1.420	4.144	24.7	0.957	2.603
	Folate (μg)	27.2	269	751	28.4	174	528
	Vitamin B12 (μg)	16.1	14.04	27.44	26.4	10.87	20.95
	Vitamin C (mg)	23.4	56.5	110.5	13.3	40.9	74.3
	Vitamin A (μg)	13.8	1145	2027	12.9	923	1411
	Vitamin D (IU)	15.1	83	154	11.5	68	110
	Vitamin E (mg)	21.3	12.43	34.35	13.7	10.14	21.53
**Minerals**	Calcium (mg)	23.2	1492	3105	31.1	1111	2262
	Copper (mg)	30.5	1.691	5.292	32.7	1.315	3.584
	Iron (mg)	29.6	17.89	57.26	28.4	12.86	39.53
	Magnesium (mg)	31.6	305	1028	38.1	233	688
	Manganese (mg)	36.6	3.519	12.357	40.4	2.655	8.617
	Phosphorus (mg)	30.6	1955	5677	38.8	1313	3724
	Potassium (mg)	24.5	3182	9506	34.1	2382	6601
	Zinc (mg)	25.5	24.32	66.27	39.0	17.68	47.21

% Deviance Explained = (1 - Residual Deviance / Null Deviance) * 100. $p<0.001$ for Chi-square residual deviance tests of goodness of fit for all food groups and nutrients. Mean total daily household consumption estimates from each household survey are provided for better interpretability of mean absolute error. Abbreviations: FCS-HH (2013 Food Consumption Survey), HSES-HH (2012/2014 Household Socio-Economic Survey), MAE (mean absolute error), HH (household), IU (international unit; 40 IU = 1 μg). Statistics are survey-weighted.

Table S7. Mean bias of household disaggregation methods in estimating ranks of food group and nutrient intakes and intake densities (per 100 kcal) across 14 age-sex groups (Aims 2 and 3)

	**Validation Metric:**	**Mean Rank Bias in Intake**						**Mean Rank Bias in Intake Density (per 100 kcal)**					
	**Household Survey:**	**FCS-HH (n=1012**			**HSES-HH (n=9424)**			**FCS-HH (n=1012**			**HSES-HH (n=9424)**		
	**Disaggregation Method:**	**SD1**	**SD2**	**AME**	**SD1**	**SD2**	**AME**	**SD1**	**SD2**	**AME**	**SD1**	**SD2**	**AME**
**Food Groups**	Animal fat, eggs, and dairy products (g)	5.1	5.0	5.1	4.4	5.0	5.0	3.9	3.6	4.6	4.3	4.6	5.3
	Baked and fried flour products (g)	5.6	6.0	4.7	5.7	5.7	5.4	4.0	4.1	3.3	4.9	4.6	4.6
	Flours, grains, and noodles (g)	3.9	4.4	2.9	3.4	5.4	4.7	3.4	4.1	2.3	1.6	3.0	1.9
	Fruits and non-tuber vegetables (g)	5.6	5.6	5.6	5.6	5.7	6.3	5.3	4.9	3.7	3.1	3.1	4.6
	Meat, fish, and poultry (g)	5.1	5.1	3.6	3.6	4.6	4.4	5.6	5.6	4.6	3.1	3.3	2.9
	Milk (except fermented) (g)	2.7	3.1	3.0	2.0	2.3	2.7	1.6	1.7	3.0	3.6	4.0	4.0
	Salt (g)	4.1	4.0	2.4	4.9	4.9	4.6	3.6	3.6	3.4	5.3	5.3	3.6
	Starchy root vegetables (g)	4.9	5.1	5.0	3.6	4.7	5.4	4.9	4.4	3.1	2.4	2.4	2.1
	Sugar and sweeteners (g)	3.9	4.3	4.0	4.9	5.3	5.3	4.3	4.3	4.3	4.0	4.4	6.1
	Tea or coffee (solid equivalent) (g)	5.0	4.7	4.3	4.3	4.3	3.7	4.4	4.4	4.4	3.6	3.4	4.6
	Vegetable oils (any) (g)	4.6	5.0	4.6	2.0	3.4	4.0	4.1	4.3	4.1	3.4	3.3	4.1
**Macronutrients**	Energy (kcal)	4.0	4.3	3.1	4.7	5.3	4.7	N/A	N/A	N/A	N/A	N/A	N/A
	Carbohydrates (g)	3.3	4.1	3.3	4.6	5.3	4.7	3.4	3.3	3.9	4.7	4.0	3.6
	Protein (g)	5.0	5.0	4.0	4.7	5.1	5.0	4.6	4.3	3.3	3.0	3.0	4.1
	Total fat (g)	5.7	5.4	4.1	4.9	4.9	5.0	4.1	4.6	4.9	4.6	4.0	4.1
	Alcohol (g)	3.3	3.1	2.9	2.2	2.1	3.1	3.4	3.1	4.3	1.9	1.8	2.1
	Water (g)	5.9	5.6	4.6	5.1	5.0	4.7	4.4	4.4	4.0	3.7	3.4	3.9
	Fiber (g)	3.6	4.1	3.1	4.1	4.9	4.3	4.6	4.0	5.0	5.9	5.0	5.3
	Phytosterols (mg)	4.7	4.9	3.4	4.9	5.1	4.6	5.1	5.6	6.0	4.7	4.4	4.9
**Vitamins**	Thiamin (mg)	4.3	4.7	3.7	4.7	4.7	4.3	3.4	3.4	4.3	3.7	3.3	4.0
	Riboflavin (mg)	6.0	6.0	4.7	4.7	4.9	4.7	3.0	3.1	3.1	4.4	4.4	3.7
	Niacin (mg)	5.0	5.0	3.6	4.1	4.9	4.3	6.4	6.3	6.3	5.1	4.6	5.7
	Pantothenic acid (mg)	5.6	5.4	4.1	4.9	4.9	4.9	5.0	5.0	4.0	4.0	4.0	5.1
	Vitamin B6 (mg)	4.6	4.6	3.6	4.7	4.7	4.4	6.0	5.7	5.0	5.1	4.9	4.0
	Folate (µg)	4.3	4.7	3.4	4.7	5.0	4.7	6.0	5.6	4.9	5.0	5.3	3.6
	Vitamin B12 (µg)	6.6	6.6	3.9	5.0	4.9	4.3	6.7	6.7	5.9	4.9	4.9	4.6
	Vitamin C (mg)	5.7	5.7	5.3	4.9	5.7	6.3	4.6	5.3	4.9	3.4	3.1	4.6
	Vitamin A (µg)	6.6	6.7	3.9	5.3	5.3	4.1	6.9	7.0	6.0	6.4	6.4	6.0
	Vitamin D (IU)	4.7	4.7	4.4	5.9	5.9	4.7	4.3	4.4	3.9	4.9	5.0	4.7
	Vitamin E (mg)	4.6	5.3	4.0	3.4	4.6	4.0	6.1	5.7	6.0	6.3	6.1	5.7
**Minerals**	Calcium (mg)	6.6	6.4	5.9	6.1	6.0	5.9	3.1	2.6	3.9	4.1	4.3	3.9
	Copper (mg)	4.6	5.1	2.7	4.1	5.1	4.3	6.0	6.0	6.3	5.6	6.0	5.3
	Iron (mg)	3.9	4.6	2.9	4.7	4.6	4.6	5.6	5.1	5.7	4.6	4.4	4.6
	Magnesium (mg)	4.7	5.0	4.1	5.1	5.3	5.0	3.4	3.1	3.4	2.9	3.0	3.3
	Manganese (mg)	3.6	4.1	3.1	4.9	5.0	4.6	4.9	4.6	6.0	5.1	4.6	5.6
	Phosphorus (mg)	5.4	5.3	4.7	5.1	5.1	5.0	1.7	1.6	3.1	4.0	4.3	4.3
	Potassium (mg)	5.0	5.0	4.7	5.1	5.1	5.1	3.3	3.4	4.3	3.6	4.1	4.0
	Zinc (mg)	5.3	5.0	4.1	4.7	5.0	5.0	4.6	4.3	4.3	3.0	2.9	4.4

Green-Yellow-Red shading indicates the magnitude of mean bias in estimated ranks of intake (Green: minimum observed mean rank bias; Yellow: median; red: maximum), and Blue-Yellow-Red shading indicates magnitude of mean bias in estimated ranks of intake density (per 100 kcal) (Blue: minimum observed absolute mean rank bias; Yellow: median; Red: maximum). Abbreviations: FCS-HH (2013 Food Consumption Survey), HSES-HH (2012/2014 Household Socio-Economic Survey), SD1 (unadjusted statistical disaggregation method), SD2 (AME-like statistical disaggregation method), IU (international unit; 40 IU = 1 μg). Statistics are survey weighted.

Table S8. In-sample fit statistics for increasingly complex predictive models of individuals' dietary intakes and intake densities (per 100 kcal) in the FCS-24 (Aim 4)

	**Measurement Type:**											**Nutrient Intake**								
	**Validation Metric:**	**1 / (Residual Deviance / Null Deviance) * 100**							**Mean Absolute Error**											
	**Model Designation:**	**1**	**2**	**3**	**4a**	**4b**	**4c**	**5**	**1**	**2**	**3**	**4a**	**4b**	**4c**	**5**	**(SD1)**	**(SD2)**	**(AME)**	**Intake**
**Macronutrients**	Energy (kcal)	53.6	52.9	51.9	59.3	66.0	68.3	71.8	229	231	229	209	191	185	178	384	1344	1095	1864
	Carbohydrates (g)	44.7	45.7	44.8	52.4	60.2	67.4	62.4	35.65	35.58	35.92	32.63	30.28	27.49	29.57	56.58	136.63	126.35	241.10
	Protein (g)	54.1	55.3	56.7	60.8	68.2	69.7	71.5	8.83	8.67	8.75	8.47	7.25	7.12	7.01	20.85	65.01	50.15	70.09
	Total fat (g)	40.8	42.5	45.8	50.1	55.0	64.9	65.3	9.92	9.86	9.62	9.49	8.40	7.77	7.72	23.12	69.22	47.73	66.38
	Alcohol (g)	91.9	92.0	94.6	94.6	95.4	99.1	99.0	0.73	0.68	0.33	0.30	0.41	0.30	0.31	1.60	1.58	1.62	1.47
	Water (g)	29.0	34.1	30.0	43.4	48.0	61.7	64.1	133.46	129.52	129.21	116.17	110.92	99.64	95.32	255.61	725.61	572.34	572.27
	Fiber (g)	48.8	48.1	48.2	56.0	61.6	71.5	72.8	1.1	1.1	1.1	1.0	1.0	0.9	0.9	2.3	6.5	5.8	8.6
	Phytosterols (mg)	56.3	55.4	53.5	60.1	68.0	75.0	75.7	50	51	51	47	42	40	42	140	316	262	424
**Vitamins**	Thiamin (mg)	37.9	37.9	39.6	47.2	54.8	62.4	63.5	0.107	0.108	0.104	0.097	0.088	0.084	0.085	0.252	0.727	0.567	0.784
	Riboflavin (mg)	31.0	35.7	31.8	40.5	47.0	70.2	71.8	0.207	0.196	0.201	0.181	0.174	0.137	0.141	0.468	1.266	1.027	1.220
	Niacin (mg)	57.2	58.5	60.2	64.5	67.3	74.1	75.4	1.394	1.389	1.352	1.273	1.187	1.135	1.162	4.066	13.031	9.522	13.064
	Pantothenic acid (mg)	37.6	37.7	38.5	44.9	52.2	66.1	69.6	0.443	0.450	0.458	0.430	0.392	0.334	0.334	1.087	3.143	2.421	3.111
	Vitamin B6 (mg)	44.7	44.8	46.1	52.0	62.2	69.8	69.7	0.103	0.104	0.099	0.094	0.084	0.075	0.079	0.235	0.692	0.542	0.628
	Folate (µg)	41.1	40.2	38.2	48.3	52.9	72.4	72.4	25	25	24	22	22	17	17	37	100	81	132
	Vitamin B12 (µg)	67.4	68.7	71.9	73.2	76.1	90.5	91.5	1.10	0.96	1.06	1.04	0.95	0.61	0.61	2.96	4.44	3.00	6.35
	Vitamin C (mg)	42.5	42.1	46.5	49.8	56.1	83.4	83.2	1.5	1.4	1.4	1.3	1.2	0.8	0.8	7.2	24.3	20.8	12.4
	Vitamin A (µg)	59.0	62.7	63.8	65.3	67.7	90.1	88.0	109	95	95	95	97	53	59	227	389	266	448
	Vitamin D (IU)	31.0	34.6	31.7	36.0	46.5	77.4	78.1	6	6	6	6	6	4	4	13	33	24	26
	Vitamin E (mg)	28.2	27.7	29.1	57.3	63.0	64.5	70.0	0.97	0.98	0.96	0.75	0.69	0.67	0.65	1.73	5.35	4.39	5.28
**Minerals**	Calcium (mg)	29.2	34.2	33.9	50.1	55.0	68.7	68.5	112	108	109	95	89	74	78	227	569	483	432
	Copper (mg)	58.6	59.8	60.4	62.7	67.7	85.2	84.7	0.152	0.153	0.149	0.138	0.135	0.097	0.101	0.325	0.681	0.550	0.986
	Iron (mg)	49.4	47.8	51.9	55.4	61.5	68.7	68.6	1.34	1.35	1.29	1.27	1.15	1.10	1.12	2.59	7.60	5.98	10.03
	Magnesium (mg)	49.8	49.8	48.3	57.9	62.1	69.2	70.5	23	23	24	21	20	18	18	51	143	116	168
	Manganese (mg)	52.6	52.4	50.7	61.1	65.0	70.6	72.1	0.292	0.295	0.297	0.263	0.249	0.239	0.240	0.495	1.337	1.239	2.172
	Phosphorus (mg)	52.5	54.2	52.9	58.9	62.3	70.6	69.2	120	117	117	109	104	97	99	279	846	667	907
	Potassium (mg)	39.0	39.0	36.9	43.2	48.7	63.8	63.7	205	204	210	199	182	160	159	491	1657	1223	1436
	Zinc (mg)	61.9	63.2	62.7	67.9	71.8	73.2	74.7	1.14	1.17	1.15	1.07	0.99	0.98	0.99	3.40	11.30	7.88	10.85
	**Measurement Type:**											**Nutrient Intake Density (per 100 kcal)**								
	**Validation Metric:**	**1 / (Residual Deviance / Null Deviance) * 100**							**Mean Absolute Error**											
	**Model Designation:**	**1**	**2**	**3**	**4a**	**4b**	**4c**	**5**	**1**	**2**	**3**	**4a**	**4b**	**4c**	**5**	**(SD1)**	**(SD2)**	**(AME**	**Densit**

																	)	y
**Macronutrients**	Energy (kcal)	N/A	N/A	N/A	N/A	N/A	N/A	N/A	N/A	N/A	N/A	N/A	N/A	N/A	N/A	N/A	N/A	N/A	N/A
	Carbohydrates (g)	6.7	11.1	9.8	24.0	35.3	47.3	45.9	0.856	0.856	0.853	0.798	0.734	0.697	0.699	1.358	1.575	1.135	12.920
	Protein (g)	13.6	14.7	14.4	26.3	39.5	51.8	52.8	0.292	0.296	0.297	0.283	0.240	0.228	0.235	0.418	0.517	0.370	3.777
	Total fat (g)	23.2	25.0	25.7	34.9	48.4	61.5	57.4	0.291	0.285	0.287	0.269	0.243	0.226	0.225	0.569	0.703	0.378	3.574
	Alcohol (g)	88.3	88.5	91.9	92.4	92.6	97.4	96.8	0.029	0.031	0.020	0.019	0.017	0.014	0.014	0.068	0.066	0.064	0.067
	Water (g)	14.2	18.7	12.9	24.4	35.4	59.1	59.3	6.566	6.496	6.620	6.373	5.940	4.608	4.513	8.816	11.706	11.055	31.081
	Fiber (g)	27.6	31.7	30.7	36.1	47.0	67.7	65.6	0.04	0.04	0.04	0.03	0.03	0.03	0.03	0.08	0.06	0.10	0.47
	Phytosterols (mg)	16.7	17.6	18.9	27.2	37.9	69.8	64.9	2.0	2.0	2.1	2.0	1.8	1.4	1.6	5.0	3.5	3.3	22.9
**Vitamins**	Thiamin (mg)	17.7	19.1	19.3	32.1	39.0	61.2	65.0	0.0039	0.0039	0.0040	0.0037	0.0034	0.0028	0.0028	0.0062	0.0063	0.0065	0.0426
	Riboflavin (mg)	11.6	13.9	13.5	25.8	40.2	71.0	76.0	0.0096	0.0096	0.0095	0.0089	0.0088	0.0061	0.0053	0.0138	0.0163	0.0122	0.0661
	Niacin (mg)	40.5	41.8	39.5	45.7	52.9	67.2	70.9	0.0612	0.0619	0.0630	0.0578	0.0545	0.0438	0.0452	0.1236	0.1432	0.1170	0.7093
	Pantothenic acid (mg)	16.6	13.4	12.7	23.7	38.4	63.8	70.5	0.0191	0.0191	0.0195	0.0183	0.0169	0.0129	0.0116	0.0292	0.0327	0.0314	0.1686
	Vitamin B6 (mg)	43.6	45.6	47.1	51.9	54.3	71.4	71.7	0.0047	0.0047	0.0047	0.0044	0.0044	0.0033	0.0034	0.0093	0.0102	0.0101	0.0342
	Folate (µg)	17.1	19.8	20.3	28.2	32.8	66.2	65.8	0.9	0.9	0.9	0.8	0.8	0.6	0.6	1.0	1.0	1.0	7.1
	Vitamin B12 (µg)	57.4	59.7	57.4	61.3	68.7	85.5	86.3	0.057	0.057	0.058	0.053	0.046	0.036	0.036	0.137	0.131	0.091	0.339
	Vitamin C (mg)	55.6	55.0	55.1	59.2	64.5	79.8	79.4	0.11	0.11	0.11	0.11	0.10	0.08	0.08	0.35	0.56	0.60	0.70
	Vitamin A (µg)	53.3	57.3	56.9	59.6	60.9	87.8	86.2	5.3	5.1	5.0	4.7	4.8	2.8	3.1	11.0	11.5	8.4	23.7
	Vitamin D (IU)	42.1	46.7	41.2	46.1	54.9	82.2	80.3	0.3	0.3	0.3	0.3	0.3	0.2	0.2	0.6	0.7	0.4	1.4
	Vitamin E (mg)	9.7	13.0	7.7	39.5	50.3	62.1	57.7	0.043	0.043	0.042	0.034	0.032	0.028	0.031	0.057	0.069	0.091	0.286
**Minerals**	Calcium (mg)	28.1	32.9	33.8	48.4	57.4	73.4	72.7	5.6	5.6	5.5	4.8	4.5	3.6	3.6	7.8	9.4	7.9	23.6
	Copper (mg)	47.0	50.9	48.8	50.8	54.5	84.6	85.0	0.0068	0.0074	0.0077	0.0071	0.0078	0.0041	0.0043	0.0151	0.0133	0.0129	0.0528
	Iron (mg)	14.7	14.9	16.1	24.9	33.2	63.7	64.0	0.045	0.045	0.045	0.043	0.040	0.030	0.032	0.056	0.054	0.059	0.541
	Magnesium (mg)	21.1	21.1	21.7	27.3	27.8	60.8	56.3	0.6	0.7	0.7	0.7	0.6	0.5	0.5	1.0	1.0	1.1	9.1
	Manganese (mg)	41.3	43.1	44.6	49.1	55.6	64.6	66.9	0.0093	0.0091	0.0097	0.0086	0.0086	0.0072	0.0071	0.0146	0.0149	0.0200	0.1171
	Phosphorus (mg)	31.8	34.1	32.6	43.5	48.9	70.0	70.3	3.6	3.6	3.7	3.3	3.2	2.5	2.5	4.9	6.5	4.7	48.9
	Potassium (mg)	16.8	16.6	16.5	22.5	32.9	54.2	54.5	8.6	8.6	8.8	8.5	8.2	6.6	6.4	12.6	19.6	15.7	78.1
	Zinc (mg)	24.9	24.6	23.8	35.2	42.5	56.9	49.7	0.053	0.053	0.053	0.048	0.045	0.041	0.044	0.099	0.126	0.076	0.587

See Table 4 for detailed descriptions of models 1-5. Brief description of variable categories considered for selection in each model: (1) Household and individual demographic, socioeconomic, and lifestyle characteristics, (2) Model 1 variables + quantitative total household consumption of food groups and nutrients, (3) Model 2 variables + individuals' self-evaluation of nutrition knowledge and its application to their lives, (4a) Model 3 variables + cursory qualitative 24-hour recall and assessment of eating behaviors, (4b) Model 3 variables + cursory semiquantitative 24-hour recall and assessment of eating behaviors, (4c) Model 3 variables + detailed semiquantitative 24-hour recall, (5) Model 4 variables + measured

anthropometry. For comparison with mean absolute error of prediction models 1-5, mean absolute error of unadjusted and AME-like statistical disaggregation and adult male equivalent methods applied to the FCS-HH are provided in columns “(SD1)”, "(SD2)", and “(AME)”, respectively. Mean dietary intake and intake density from the FCS-24 are also provided in the "Intake" and "Density" columns for better interpretability of all mean absolute error estimates. Green-Yellow-Red shading indicates the magnitude of mean absolute error in predicting dietary nutrient intake proportional to mean observed dietary intake (Green: minimum absolute error; Yellow: median; Red: maximum) and Blue-Yellow-Red shading indicates the magnitude of mean absolute error in predicting dietary nutrient intake density proportional to mean observed dietary intake density (per 100 kcal) (Blue: minimum absolute error; Yellow: median; Red: maximum). Abbreviations: FCS-HH (2013 Food Consumption Survey), FCS-24 (nested 24-hour recall), IU (international unit; 40 IU = 1 μg).

Table S9. Mean absolute error of alternate prediction methods of individuals' dietary intake densities of nutrients in the FCS-24: direct prediction of nutrient densities (left) vs. estimation based on separate prediction of nutrient intake and energy intake (right) (Aim 4)

	**Prediction Method:**	**Direct Prediction of Nutrient Intake Densities**							**Separate Prediction of Nutrient and Energy Intake**							
	**Model Designation:**	**1**	**2**	**3**	**4a**	**4b**	**4c**	**5**	**1**	**2**	**3**	**4a**	**4b**	**4c**	**5**	**Density**
**Macronutrients**	Energy (kcal)	N/A	N/A	N/A	N/A	N/A	N/A	N/A	N/A	N/A	N/A	N/A	N/A	N/A	N/A	N/A
	Carbohydrates (g)	0.856	0.856	0.853	0.798	0.734	0.697	0.699	0.833	0.879	0.814	0.784	0.814	0.733	0.728	12.920
	Protein (g)	0.292	0.296	0.297	0.283	0.240	0.228	0.235	0.279	0.284	0.280	0.271	0.262	0.229	0.229	3.777
	Total fat (g)	0.291	0.285	0.287	0.269	0.243	0.226	0.225	0.288	0.280	0.277	0.274	0.273	0.215	0.212	3.574
	Alcohol (g)	0.029	0.031	0.020	0.019	0.017	0.014	0.014	0.029	0.032	0.018	0.019	0.018	0.012	0.016	0.067
	Water (g)	6.566	6.496	6.620	6.373	5.940	4.608	4.513	6.292	6.100	6.289	5.977	5.718	4.529	4.441	31.081
	Fiber (g)	0.04	0.04	0.04	0.03	0.03	0.03	0.03	0.04	0.04	0.04	0.03	0.03	0.03	0.03	0.47
	Phytosterols (mg)	2.0	2.0	2.1	2.0	1.8	1.4	1.6	1.9	1.9	2.1	1.9	1.8	1.6	1.8	22.9
**Vitamins**	Thiamin (mg)	0.0039	0.0039	0.0040	0.0037	0.0034	0.0028	0.0028	0.0038	0.0037	0.0038	0.0034	0.0033	0.0028	0.0027	0.0426
	Riboflavin (mg)	0.0096	0.0096	0.0095	0.0089	0.0088	0.0061	0.0053	0.0090	0.0090	0.0090	0.0086	0.0083	0.0062	0.0057	0.0661
	Niacin (mg)	0.0612	0.0619	0.0630	0.0578	0.0545	0.0438	0.0452	0.0615	0.0610	0.0615	0.0600	0.0557	0.0462	0.0469	0.7093
	Pantothenic acid (mg)	0.0191	0.0191	0.0195	0.0183	0.0169	0.0129	0.0116	0.0179	0.0177	0.0181	0.0176	0.0169	0.0138	0.0125	0.1686
	Vitamin B6 (mg)	0.0047	0.0047	0.0047	0.0044	0.0044	0.0033	0.0034	0.0046	0.0045	0.0046	0.0044	0.0041	0.0035	0.0035	0.0342
	Folate (µg)	0.9	0.9	0.9	0.8	0.8	0.6	0.6	0.9	0.9	0.9	0.8	0.9	0.6	0.6	7.1
	Vitamin B12 (µg)	0.057	0.057	0.058	0.053	0.046	0.036	0.036	0.061	0.059	0.060	0.054	0.051	0.036	0.038	0.339
	Vitamin C (mg)	0.11	0.11	0.11	0.11	0.10	0.08	0.08	0.11	0.11	0.11	0.11	0.10	0.08	0.08	0.70
	Vitamin A (µg)	5.3	5.1	5.0	4.7	4.8	2.8	3.1	5.4	4.9	5.0	4.7	4.6	3.0	3.2	23.7
	Vitamin D (IU)	0.3	0.3	0.3	0.3	0.3	0.2	0.2	0.3	0.3	0.3	0.3	0.3	0.2	0.2	1.4
	Vitamin E (mg)	0.043	0.043	0.042	0.034	0.032	0.028	0.031	0.042	0.041	0.041	0.033	0.032	0.030	0.031	0.286
**Minerals**	Calcium (mg)	5.6	5.6	5.5	4.8	4.5	3.6	3.6	5.4	5.3	5.5	4.7	4.6	3.6	3.6	23.6
	Copper (mg)	0.0068	0.0074	0.0077	0.0071	0.0078	0.0041	0.0043	0.0074	0.0074	0.0076	0.0071	0.0070	0.0048	0.0045	0.0528
	Iron (mg)	0.045	0.045	0.045	0.043	0.040	0.030	0.032	0.042	0.042	0.042	0.041	0.040	0.030	0.031	0.541
	Magnesium (mg)	0.6	0.7	0.7	0.7	0.6	0.5	0.5	0.6	0.6	0.6	0.6	0.6	0.5	0.5	9.1
	Manganese (mg)	0.0093	0.0091	0.0097	0.0086	0.0086	0.0072	0.0071	0.0093	0.0093	0.0096	0.0094	0.0082	0.0080	0.0085	0.1171
	Phosphorus (mg)	3.6	3.6	3.7	3.3	3.2	2.5	2.5	3.5	3.4	3.5	3.2	3.4	2.5	2.9	48.9
	Potassium (mg)	8.6	8.6	8.8	8.5	8.2	6.6	6.4	8.2	8.2	8.4	8.2	8.1	6.2	6.2	78.1
	Zinc (mg)	0.053	0.053	0.053	0.048	0.045	0.041	0.044	0.051	0.051	0.051	0.047	0.045	0.042	0.042	0.587

Columns under "Separate Prediction of Nutrient and Energy Intake" present mean absolute error of nutrient intake densities estimated by separately predicting nutrient intake and energy intake, dividing predicted nutrient intake by predicted energy intake, and comparing the results to observed dietary intake measurements in the FCS-24. Columns under "Direct Prediction of Nutrient Intake Densities" are reproduced from Table 3.8 for comparison. See Table 4 for detailed descriptions of models 1-5. Brief description of variable categories considered for selection in each model: (1) Household and individual demographic, socioeconomic, and lifestyle characteristics, (2) Model 1 variables + quantitative total household consumption of food groups and nutrients, (3) Model 2 variables + individuals' self-evaluation of nutrition knowledge and its application to their lives, (4a) Model 3 variables + cursory qualitative 24-hour recall and assessment of eating behaviors, (4b) Model 3 variables + cursory semiquantitative 24-hour recall and assessment of eating behaviors, (4c) Model 3 variables + detailed semiquantitative 24-hour recall, (5) Model 4 variables + measured anthropometry. Mean dietary intake density from the FCS-24 is provided in the "Density" column for better interpretability of

all mean absolute error estimates. Blue-Yellow-Red shading indicates the magnitude of mean absolute error in predicting dietary nutrient intake density proportional to mean observed dietary intake density (per 100 kcal) (Blue: minimum absolute error; Yellow: median; Red: maximum). Abbreviations: FCS-24 (nested 24-hour recall of the 2013 Food Consumption Survey), IU (international unit; 40 IU = 1 μg).
